# Rapid, tunable, and multiplexed detection of RNA using convective array PCR

**DOI:** 10.1038/s42003-023-05346-4

**Published:** 2023-09-23

**Authors:** Andrew T. Sullivan, Vibha Rao, Tyler Rockwood, Jahnavi Gandhi, Sarah Gruzka, Logan O’Connor, Bonnie Wang, Katherine B. Ragan, David Yu Zhang, Dmitriy Khodakov

**Affiliations:** Torus Biosystems, Inc., Medford, MA USA

**Keywords:** Biological techniques, Biotechnology

## Abstract

Detection of RNA targets is typically achieved through RT-qPCR or RNAseq. RT-qPCR is rapid but limited in number and complexity of targets detected, while RNAseq is high-throughput but takes multiple days. We demonstrate simultaneous amplification and detection of 28 distinct RNA targets from a single unsplit purified RNA sample in under 40 minutes using our convective array PCR (caPCR) technology. We integrate tunable strand displacement probes into caPCR to allow detection of RNA species with programmable sequence selectivity for either a single, perfectly matched target sequence or for targets with up to 2 single-nucleotide variants within the probe-binding regions. Tunable probes allow for robust detection of desired RNA species against high homology background sequences and robust detection of RNA species with significant sequence diversity due to community-acquired mutations. As a proof-of-concept, we experimentally demonstrated detection of 7 human coronaviruses and 7 key variants of concern of SARS-CoV-2 in a single assay.

## Abstract (web summary)

Integrating tunable strand displacement probes into a convective array PCR platform allows simultaneous amplification and detection of 28 distinct RNA targets from a single sample in under 40 minutes.

## Introduction

In molecular biology, RNA targets are typically detected and quantitated by reverse-transcription quantitative polymerase chain reaction (RT-qPCR) or high-throughput RNA sequencing (RNAseq). RT-qPCR is one of the most widely used tools for assessing RNA targets due to its rapid turn-around time and sensitive limit of detection. Despite its utility, standard RT-qPCR suffers from low target multiplexing (often 5 targets or less for fluorescence-based readouts) and lack of single-nucleotide specificity in detection^1,2^. Alternatively, RNAseq can be used to probe RNA targets with massive multiplexing and increased sensitivity for single-nucleotide variations but is labor-intensive and requires multiple days to receive results^3^.

Previously we have demonstrated the ability of our toroidal platform to improve upon traditional PCR by combining passive convective PCR with a DNA probe array (convective array PCR; caPCR)^4^. This technology reduces assay runtime from around 2 hours to approximately 30 minutes and dramatically enhances multiplexing to 10s or 100s of targets per reaction, limited only by primer compatibility and surface area available within the chamber for the probe array. Additionally, the assay takes advantage of toehold-mediated strand displacement (TMSD) probes to impart single-nucleotide specificity to the caPCR readout via the single-nucleotide-sensitive toehold-binding reaction^5,6^.

**Fig. 1:**
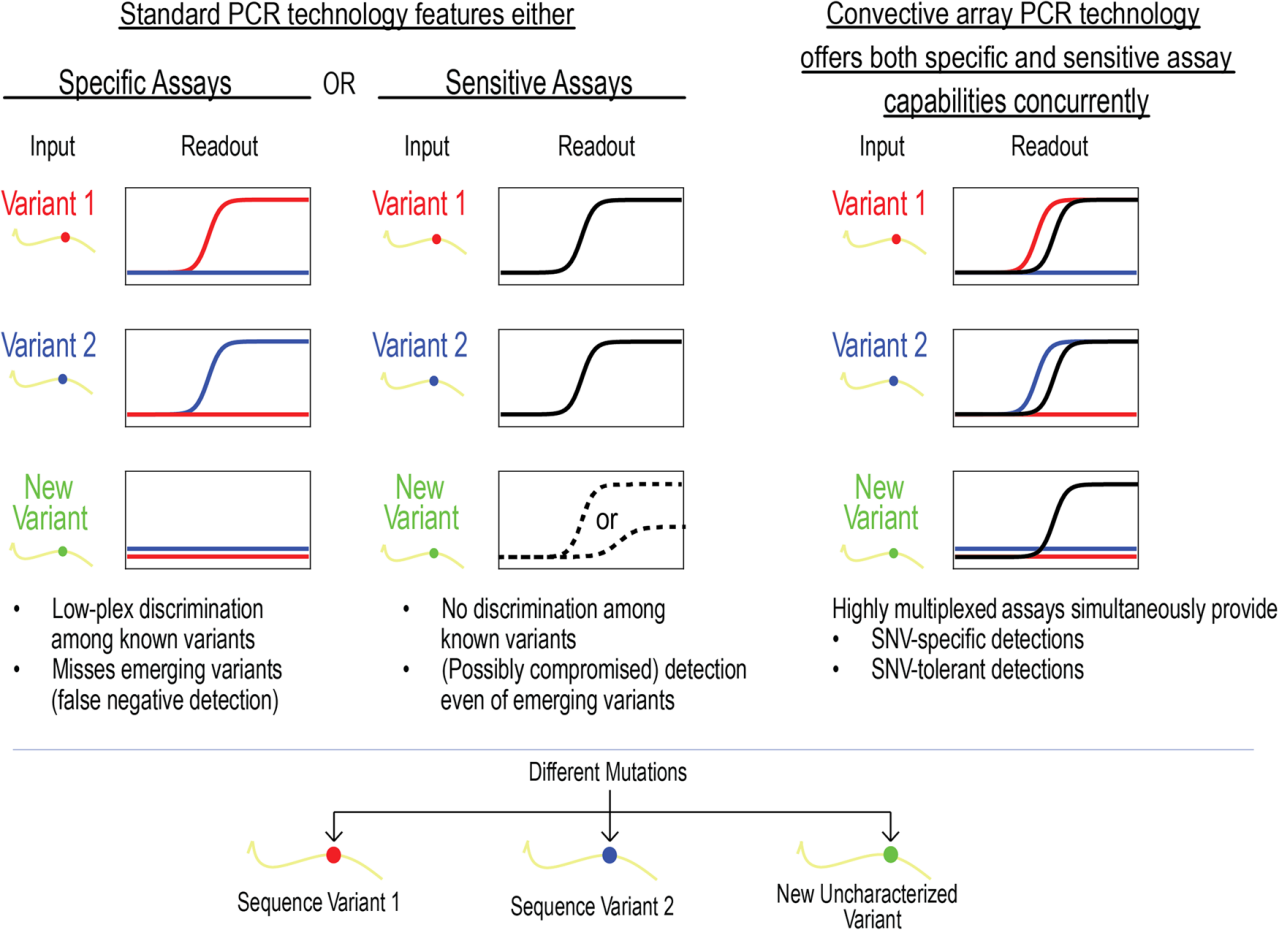
Limitations of conventional PCR assays and advantages of convective array PCR. Through careful design of the readout mechanism, conventional low-plex PCR assays can be designed to either yield high specificity or high sensitivity. In specific assays, multiple readouts may be used to discriminate amongst single-nucleotide variants (SNVs) but are unable to reliably detect targets under evolutionary pressure as new variants emerge. Sensitive assays can be designed to respond even in the presence of SNVs, providing some degree of detection even in the presence of new variants but provide no information on which variant is present. Convective array PCR (caPCR) technology leverages the benefits of both specific and sensitive detection in a single highly multiplexed assay, allowing for simultaneous SNV tracking and robust detection of evolving targets.

Although single-nucleotide resolution can provide valuable information in many contexts (including academic research in molecular biology and applications such as nucleic acid diagnostics) this level of readout may not always be optimal. Individual genetic variability or pathogen evolution can cause nucleic acid-based tests with single-nucleotide resolution to fail^7^. Thus, in nucleic acid-based testing, it is essential to have an assay that can differentiate between sequences at a single-nucleotide level when necessary while maintaining the ability to detect the presence or absence of a target sequence in the presence of mutations in the same assay (Fig. 1).

Here, we introduced a reverse-transcription (RT) step to caPCR to create a one-pot RT-caPCR assay to amplify and analyze RNA samples with newly engineered TMSD probe energetics to allow for unique tunability to impart sensitivity to target detection and specificity of target sequence as needed. The RT step is performed in the same toroidal chamber at a lower temperature to permit cDNA synthesis followed by cDNA amplification using caPCR cycling conditions. We also systematically engineered TMSD probe-based detection to achieve a range of energetic combinations that meet different functional criteria to create strand-displacement probes with programmable sequence selectivity to be either mutation-tolerant or mutation-sensitive. Single nucleotide variant (SNV)-sensitive probes allow for discrimination between targets that only differ by a single nucleotide while robust probes can detect a target even in the presence of up to 2 nucleotide mismatches within the probe region, serving as a less specific but more sensitive readout than their SNV-discriminating counterparts.

To demonstrate the utility of tunable probes in a one-pot RT-caPCR reaction, we developed a respiratory virus panel to detect the presence of each of the 7 human coronaviruses using robust probe energetics^8^. We also designed SNV-discriminatory probes targeting 5 commonly mutated sites of interest in the spike protein sequence of SARS-CoV-2, allowing for differentiation amongst 7 key variants of concern (VOCs) identified during the COVID-19 pandemic^9–11^. We tested the assay using contrived clinical samples and observed excellent sensitivity and specificity in both pathogen identification and variant discrimination. Our assay successfully detected multiple RNA targets in a single sample, indicating its ability to analyze more complex inputs. Thus, our RT-caPCR technology with tunable probes is a tool that can overcome the low multiplexing and lack of single-nucleotide specificity of standard RT-qPCR as well as slow turn-around time of RNAseq to achieve rapid, high-throughput results with single-nucleotide resolution. We anticipate this technology will have an impact beyond the diagnostic space due to its versatility and ability to achieve highly multiplexed RNA profiling traditionally only accessible via RNAseq with improved speed and the ease of traditional RT-qPCR.

## Results

### Integrating reverse transcription in caPCR

**Fig. 2:**
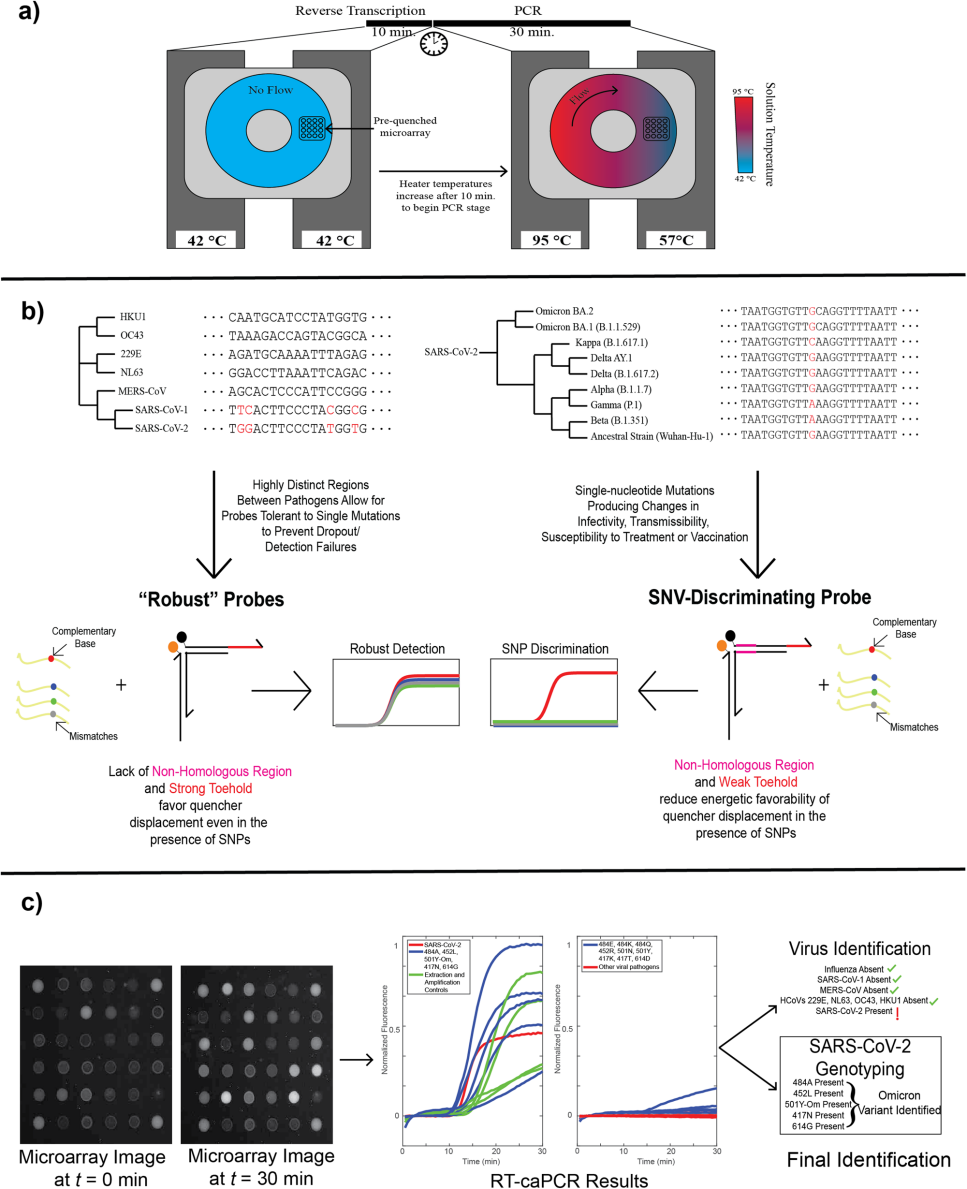
RT-caPCR workflow utilizing both robust and SNV-discriminatory probes. **a** The caPCR workflow can be extended to include a reverse transcription step (RT-caPCR) through the addition of a reverse transcriptase enzyme, and a dedicated RT stage with all device heaters set to the RT temperature (42 °C). Heater temperatures are then increased to different temperatures for denaturation (95 °C) and annealing (57 °C), inducing convective flow and PCR amplification of the cDNA templates. **b** The energetic design scheme of probes, based on DNA binding thermodynamics, can be used to address two needs in infectious disease diagnostics: I) Viral pathogen detection should be robust to small changes in the nucleotide sequence to prevent dropout if new variants arise. II) Variant discrimination requires single-nucleotide resolution. Variation of the toehold, domain, and non-homologous region (NHR) energies can be used to meet both of these requirements. **c** Example data output from a donut RT-caPCR run. Probe array images are acquired every 30 seconds for 30 minutes of caPCR. Fluorescence traces are extracted and analyzed to identify which respiratory pathogens are present and, if applicable, what variant of SARS-CoV-2 is present.

We first introduced an RT step to the standard caPCR assay. caPCR is performed using a set of 4 heaters that clamp on either face of a cartridge containing a toroidal chamber filled with the PCR reaction mixture^4^. By setting heaters on one side of the chamber to the PCR annealing temperature and the other to the denaturation temperature, a gradient is established, thereby producing a passive convective flow that drives PCR amplification. The pre-quenched toehold probe array is immobilized on the surface of the cartridge and responds through TMSD as specific PCR products are synthesized. To accommodate an initial reverse transcription step, we programmed the heaters to maintain a constant temperature of 42 °C for cDNA synthesis for 10 minutes, after which the heaters adjust to 57 °C and 95 °C respectively to initiate convective flow (Fig. 2a). A one-pot RT-PCR approach is used to reduce the number of manual handling steps in the workflow. Toehold probe-mediated detection on the caPCR platform functions optimally under asymmetric PCR conditions: an excess of the primer that produces the strand which displaces the quencher generates a stronger signal on the fluorescent probe array than a 1:1 forward-to-reverse primer ratio (Fig. S1). We ensured that this same primer also mediates cDNA synthesis from RNA targets to avoid the need to introduce separate primers for reverse transcription and PCR stages.

To develop tunable probes with programmable sequence selectivity, ranging from mutation-sensitive to mutation-tolerant, we utilized 2 sets of energetic criteria for toehold probes (Fig. 2b). Robust probes feature a stronger toehold region and a correspondingly weaker domain to reduce the overall influence of a single mutation on the initiation of the binding reaction and subsequent quencher arm displacement. For SNV discrimination, the toehold is reduced in length, and a non-homologous region (NHR) lacking complementarity to the target is introduced. This combination serves to reduce the overall energetic favorability for toehold binding and quencher arm displacement, thereby providing extra weight to single mismatches in the target sequence.

We applied RT-caPCR with both robust and SNV-discriminating probes to respiratory virus detection. Probe array images from the RT-caPCR system are processed using a custom MATLAB script to produce fluorescence traces which are then interpreted to identify both the viral target(s) present and, if applicable, the variant of SARS-CoV-2. An example is shown for the SARS-CoV-2 Omicron variant in Fig. 2c. Thus, our one-pot RT-caPCR assay demonstrates its ability to simultaneously perform both sensitive detection with robust probes as well as single-nucleotide resolution with SNV-discriminatory probes to accurately identify RNA targets of interest in a single reaction.

### Fine-tuning the energetics between robust and SNV-discriminating probes

**Fig. 3:**
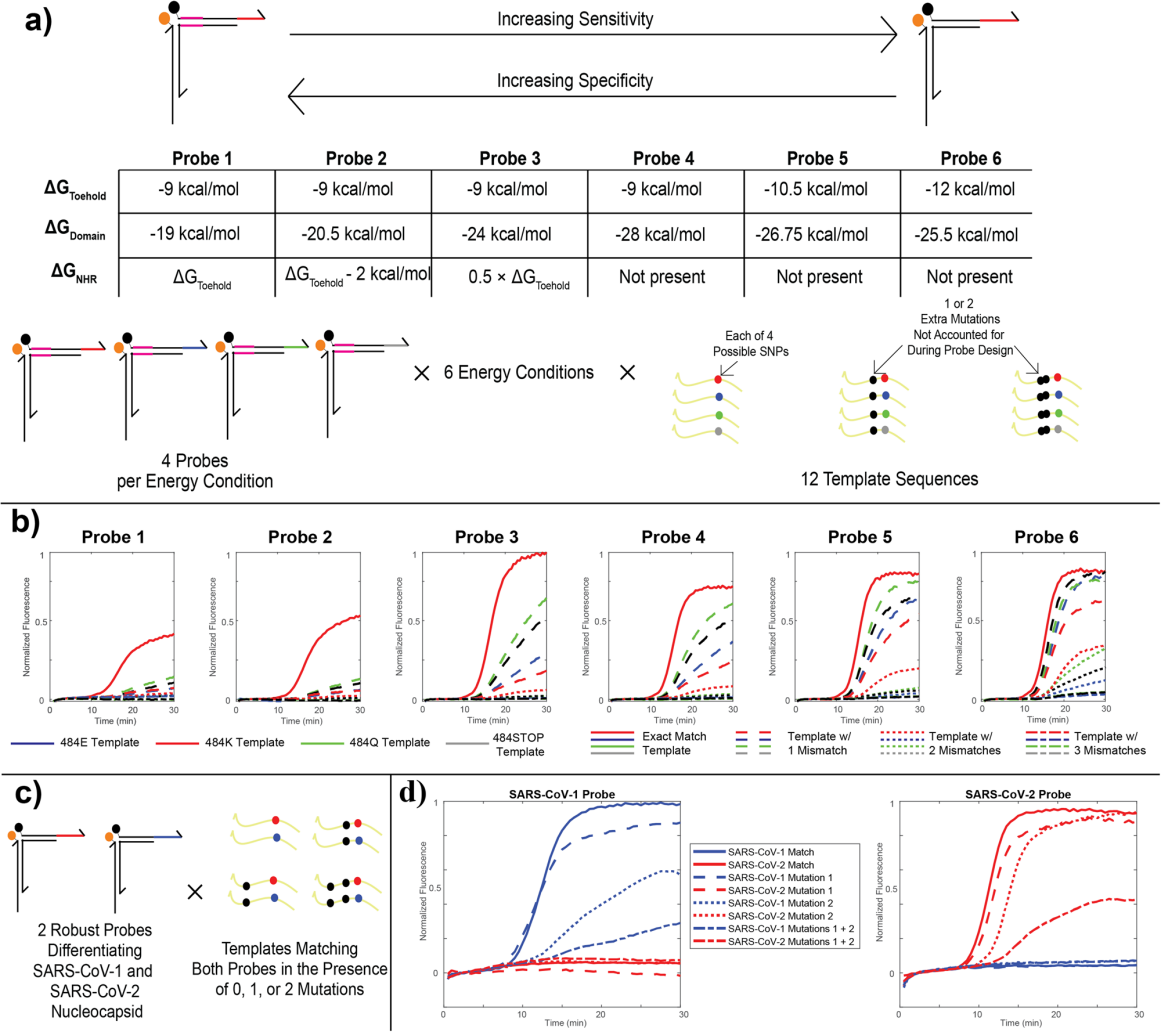
Design and validation of probe energetic schemes. **a** Design parameters for probe energetic characterization. Six different energetic schemes were designed by varying the binding energies of the toehold, domain, and NHR regions, to permit a tradeoff between single-nucleotide specificity and sensitivity in the form of robustness to mutations. Four different probes were designed for each of the six energetic designs, covering all four possible codons at the 484 mutation site in the SARS-CoV-2 spike protein, three of which have been observed in variants. Each probe was tested with 12 different plasmid templates, including the 4 unique single-nucleotide mutations alone and in the presence of one or two other mutations found further upstream in the Omicron variant. **b** caPCR results from the 484 K series of probes. Red solid curves denote the on-target performance with no mutations. Dashed, dotted, and dash-dot lines denote templates with 1, 2, or 3 mismatches relative to the probe sequence, respectively. The color specifies the codon at the 484 site (E, K, Q, or STOP). **c** Scheme for testing robust SARS-CoV-1/2 discrimination. Two probes were designed according to the energetic scheme **“Probe 5”** in part **a** for a region of the nucleocapsid gene in SARS-CoV-1 and SARS-CoV-2. Both probes were tested in the presence of the exact-match template and variants including one or both of two upstream mismatches, as well as in the presence of the exact-match template and mismatched variants of the other probe. **d** Results of the tests described in (**c**). Solid, dashed, dotted, and dash-dot lines denote exact match templates, templates with either of two SNVs, or templates with both SNVs relative to the SARS-CoV-1 (blue) or SARS-CoV-2 (red) genome.

To increase the diversity of targets detected in our RT-caPCR assay, we optimized the energetics of toehold binding and strand displacement to modulate the sensitivity-specificity tradeoff. As a case study, we used the SARS-CoV-2 ‘484E’ genotype as the template for our energetic assessment. Mutations at this site are associated with reduced neutralizing activity from host antibodies^12^, and 3 of 4 possible nucleotide bases (adenine, guanine, and cytosine) are found in the first position of the codon in naturally evolved SARS-CoV-2 variants^9^. Uracil substitution at this site would produce a stop codon and thus has not been observed in nature, but for rigor we included a thymine condition in our assessment. We designed a probe for each of the 4 possible nucleotides at this locus and established 6 different thermodynamic conditions based on the binding energy (ΔG^o^) of the 3 regions of the toehold probe (NHR, domain, and toehold; Fig. 3a, see Materials and Methods). These energetic conditions range from the most specific (Probe 1), in which the energetics of the NHR and toehold are approximately equal (around −9 kcal/mol), to the most sensitive (Probe 6), in which there is no NHR and the toehold is slightly stronger (−12 kcal/mol). In all cases, the domain binding energy is adjusted to keep the overall probe binding energy constant at approximately −40 kcal/mol.

We tested these probes with 12 different DNA templates, 4 of which represent the exact-match template for each of the 4 possible bases in the 484 codon. To demonstrate the tunability of our probe energetics, we took mutations that arose in later lineages of SARS-CoV-2 evolution and introduced them into the templates: the T478K mutation found in the Delta AY and Omicron lineages, and the S477N mutation found in Omicron lineages (schematic Fig. 3a)^10^. All 24 probes were evaluated in response to the amplification of each of the 12 templates. Figure 3b shows a subset of these results, specifically for the probes designed to target the 484 K mutation found in Beta and Gamma VOCs^10^. As the strength of the NHR is reduced and the toehold increases, detection for the exact-match template (solid red line) remains strong and the probe becomes more sensitive to templates with 1 (dashed lines) and 2 (dotted lines) mismatches. This demonstrates our ability to engineer probe energetics for very specific target-binding outcomes. Based on these results, we selected energetic scheme 2 for SNV-discriminating probes and scheme 5 for robust probes.

We designed 2 robust probes targeting the nucleocapsid gene sequence of SARS-CoV-1 and SARS-CoV-2. To ensure that these pathogen sequences could be differentiated using robust probes while remaining tolerant to mutations, we devised 8 templates containing either no mismatches, 1 of 2 mismatches, or both mismatches together (Fig. 3c, see Materials and Methods). The region selected for SARS-CoV-1 and SARS-CoV-2 detection differs between the 2 target sequences by 8 mutations: as such, mismatches in the template were selected based on these differences. As such, the SARS-CoV-2 template with 2 mismatches only has 6 differences relative to the SARS-CoV-1 sequence, thus highlighting a real-life application of the importance for precise tunability of probes for sensitivity versus specificity. Figure 3d shows the results for these tests. In all cases, we observed no cross reactivity between the SARS-CoV-1 and SARS-CoV-2 probes and templates: SARS-CoV-1 RNA was only detected by the SARS-CoV-1 probe, and SARS-CoV-2 RNA was only detected by the SARS-CoV-2 probe. The templates containing either of the 2 mutations can also be unambiguously detected by each appropriate probe, with 1 of the 2 mutations more clearly affecting performance than the other. Similarly, templates with both mutations together are still detected though performance is slightly compromised due to less energetic favorability in quencher arm displacement.

Through our targeted studies of probe energetics, we demonstrated our capacity to precisely engineer probes to have the desired tradeoff of sensitivity versus specificity. SNV-discriminatory probes have energetics that require an exact match for displacement of the quencher arm, while robust probes are more promiscuous in their binding of targets with the ability to detect an amplicon containing up to two mutations. The energetic range we explored can be applied to adjust probes as needed from energy scheme 1 to energy scheme 6 to allow for detection of targets with the desired number of mismatches from the probe sequence.

### Performance of robust and SNV-discriminatory probes

To demonstrate proof-of-concept for our highly tunable probe design, we created a full panel of both robust and SNV-discriminatory probes. Our panel highlights the utility of robust probes by screening for all 7 coronaviruses known to infect humans (HCoVs 229E, HKU1, NL63, and OC43; MERS-CoV, SARS-CoV-1, and SARS-CoV-2) as well as influenza A and B^8^. Additionally, our panel demonstrates the functionality of SNV-discriminatory probes by using 14 probes and 3 amplicons covering 5 commonly mutated sites in the SARS-CoV-2 spike gene to permit differentiation amongst variants of concern (417, 452, 484, 501, and 614; Fig. S2)^9–11^.

To assess the performance of the robust probes in our RT-caPCR assay for sensitive target detection, we performed 65 RT-caPCR tests using coronavirus-negative viral transport media (VTM) samples covering a range of patient demographics (Tables S1–S4) spiked with synthetic RNA from each of the 7 human coronaviruses, either purchased from Twist Bioscience or synthesized in-house using in vitro transcription (IVT) from gene fragments (Table S5). RNA was isolated and purified from these contrived clinical samples using a bead-based extraction method^13^ due to the precedence for using such methodology for RNA extraction in SARS-CoV-2 diagnostic workflows^14^ and was used as input to our system. This assay also included both an extraction control (RNA from MS2 bacteriophage spiked into the contrived sample prior to purification) and an amplification control (internal positive control (IPC) RNA spiked into the RT-caPCR reaction mix). Unless otherwise specified, experiments here received approximately 10^6^ copies of MS2 RNA and 10^7^ copies of viral RNA. The RT-caPCR reaction was performed in 40 minutes using 5,000 molecules of IPC control RNA, the extracted sample RNA, and 11 primer pairs covering all targets and controls.

**Fig. 4:**
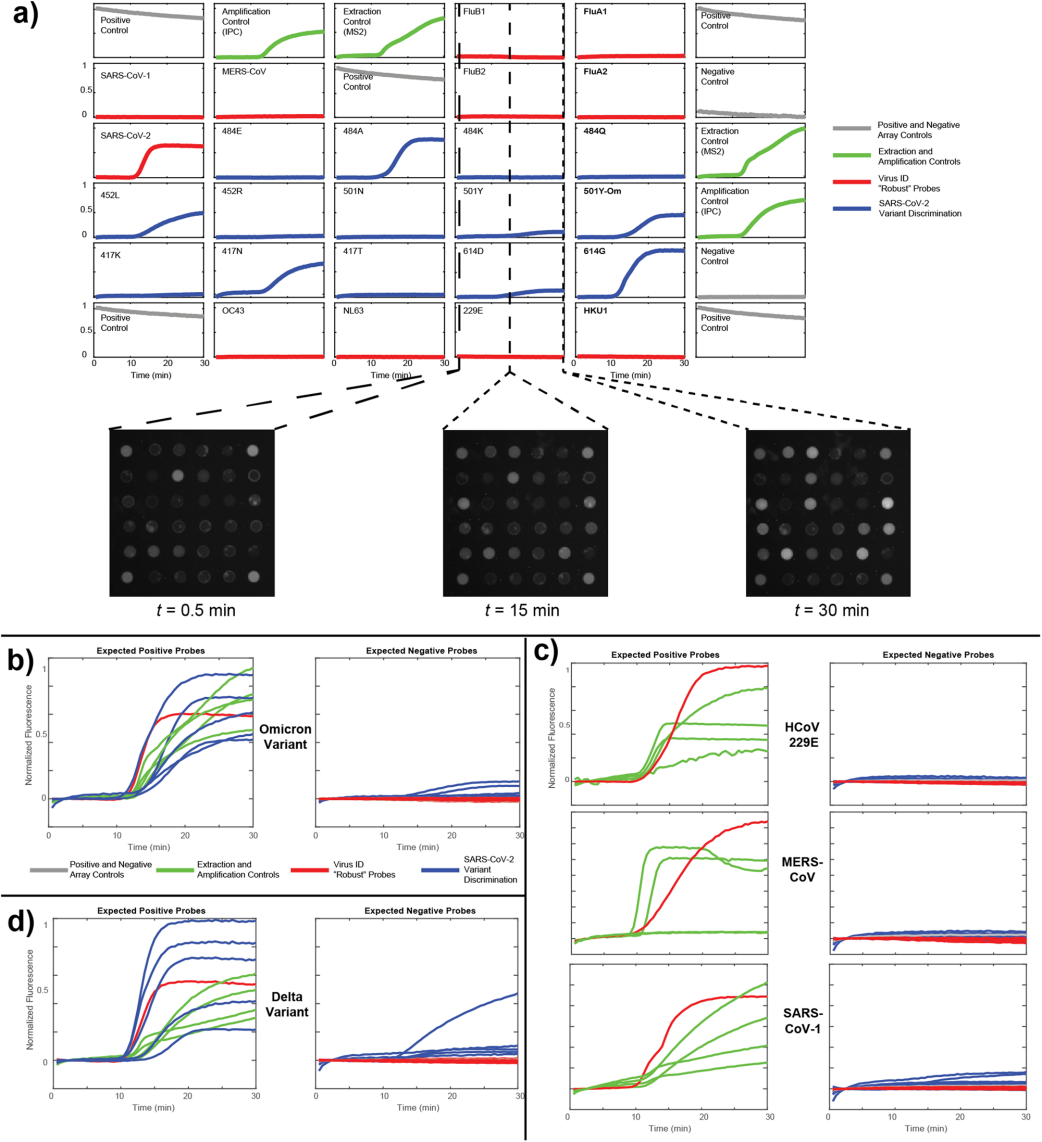
Robust and SNV-discriminatory probes function together to identify viruses and variants of SARS-CoV-2. **a** Example results from an RT-caPCR run detecting the SARS-CoV-2 Omicron variant of concern. Robust viral detection probes are in red, SNV-discriminating probes are in blue, extraction and amplification controls are in green, and positive and negative controls are in gray. Each curve represents the intensity of the corresponding probe array spot at each time point over the 60 acquired images, normalized to the brightest spot in the array. **b** Summary results corresponding to the run in **a**. All expected positives (extraction and amplification controls, on-target viral identification, and all on-target SNV-discriminating probes) are shown in the left-hand plot, whereas all expected negative probes (all other viral and SNV-discriminating probes, as well as the negative controls) are shown in the right-hand plot. **c** Viral detection results for human coronavirus 229E, Middle East Respiratory Syndrome coronavirus (MERS-CoV), and SARS-CoV-1 in the format described in (**b**). **d**. SNV-discrimination results from a SARS-CoV-2 Delta variant template in the format specified above.

RT-caPCR data were analyzed to identify the viral targets present in each sample, and, in the case of SARS-CoV-2, which specific variant was present (Fig. 4). Figure 4a shows a representative run with the SARS-CoV-2 Omicron variant as the RNA target. All robust probes for viral discrimination are shown in red while SNV-discriminating probes are shown in blue. The extraction and amplification controls are shown in green. Positive and negative probe spots correspond to fully unquenched or fully quenched probes using sequences for which no primers nor template were included and are shown in gray. This type of data can be summarized in a compressed plot as shown in Fig. 4b: all expected positive curves are depicted on the left-hand plot, and all expected negative curves are shown on the right-hand plot. While there is a small level of off-target activity on some SNV-specific probes, comparing the on-target SNV-specific probe curves (484 A, 452 L, 501Y-Om, 417 N, and 614 G) with other SNV-specific probes confirms that the on-target probes respond most strongly in all cases, as assessed by eye as well as through our fluorescence derivative-based SNV-discrimination algorithm (see Materials and Methods and Supplemental Methods). We note that, due to the Omicron variant’s high mutational load relative to other variants^11^, we designed the sequence of the 484 A probe to also include S477N and T478K mutations found in this variant to improve detection^10^. Similarly, a unique 501Y-Om probe includes Q493R, G496S, and Q498R mutations that are specifically found in the Omicron variant. A probe specific for the BA.2 subvariant of the Omicron lineage (501Y-Om2) was introduced later. This probe is identical to the 501Y-Om sequence but lacks the G496S mutation^10^.

Figure 4c shows compressed representative results from 3 runs for 3 different viral pathogen templates: human coronavirus 229E, MERS-CoV, and SARS-CoV-1. All 3 samples show clear detection of the viral RNA target with the robust pathogen identifying probes, no off-target viral identification probe activity, and the presence of all controls.

Table 1 summarizes the viral identification results from all valid trials. Competition for PCR reaction resources (e.g., polymerase, dNTPs) may suppress amplification of lower copy number targets, so an assay run was considered valid if either a viral pathogen was detected and at least 1 of the 2 controls (MS2 and IPC) was detected, or no viral pathogen was detected but both controls were detected. 11 of the samples were negative controls, which included only MS2 and IPC control RNAs. In all 11 of these samples, both IPC and MS2 were detected (100%). 36 of 36 SARS-CoV-2 samples were identified (100%) as assessed by detection of the SARS-CoV-2 nucleocapsid probe. The remaining human coronaviruses (229E, NL63, OC43, and HKU1) as well as SARS-CoV-1 and MERS-CoV targets also show high sensitivity, with 100% detection for all targets. Both the process of primer binding and amplicon-mediated quencher displacement of the probes contribute to the specificity of the panel, which was 100% across all targets. Here, specificity is calculated as the proportion of true negatives correctly detected. In short, no template induced detection at a robust probe other than the one for which it was specifically designed.

**Table 1:** Sensitivity and specificity of robust probes.

Target	Sensitivity				Specificity	
	Expected No. of Positives	No. of True Positives	%	Expected No. of Negatives	No. of True Negatives	%
SARS-CoV-2	36	36	100.00	29	29	100.00
SARS-CoV-1	3	3	100.00	62	62	100.00
MERS-CoV	3	3	100.00	62	62	100.00
HCoV-229E	3	3	100.00	62	62	100.00
HCoV-NL63	3	3	100.00	62	62	100.00
HCoV-OC43	3	3	100.00	62	62	100.00
HCoV-HKU1	3	3	100.00	62	62	100.00
IPC in Negative Extraction Controls	11	11	100.00			
MS2 in Negative Extraction Controls	11	11	100.00			

65 valid contrived human coronavirus identification runs were performed, including 11 no-virus controls with only IPC and MS2 controls, 36 SARS-CoV-2 runs, and 18 other coronavirus runs. Specificity is the percentage of runs in which the probe was correctly negative (i.e., without correct template). Sensitivity is the percentage of the runs in which the probe was correctly positive (i.e., with correct template).

Figure 4d shows a representative run highlighting the ability of our RT-caPCR assay to both identify the RNA target as well as provide single-nucleotide resolution by showing viral variant detection concurrently with pathogen identification for the SARS-CoV-2 Delta variant. While the 452 L probe shows significant off-target activity, its detection is clearly less efficient than the on-target 452 R probe (the blue curve with the highest intensity in the left-hand plot). Thus, SNV-discriminating probes allow for clear distinction between SARS-CoV-2 variants that differ only by a few single mutations.

Table 2 summarizes the performance of the SNV-specific probes for SARS-CoV-2 variants across 36 trials. The number of expected detections is equal to the number of runs for which a template displaying that mutation was included, while the number of expected negatives is the number of runs for which the corresponding template was not included. True positives are runs for which the probe of interest was the dominant SNV-specific probe for that mutation site (as assessed algorithmically), and true negatives are those runs for which it was not. In one run, we note that the 614 G probe was obscured by a foreign particle (dust), so the total for these targets is 35, rather than 36. Aside from 484E, all probes show at least 90% sensitivity. Poor performance of the 484E probe can be partially explained by the presence of a SNV (T478K mutation) in the amplicon for the Delta variants. All other probes show 100% specificity, as an amplicon will always preferentially displace its matching quencher over one with a mutation, assuming the on-target energetics are equal.

**Table 2:** Sensitivity and specificity of SNV discriminatory probes.

Probe	Sensitivity			Specificity		
	Expected No. of Positives	No. of True Positives	%	Expected No. of Negatives	No. of True Negatives	%
484E	18	14	77.78	18	18	100.00
484 A	6	6	100.00	30	30	100.00
484 K	9	9	100.00	27	27	100.00
484Q	3	3	100.00	33	33	100.00
452 L	24	24	100.00	12	12	100.00
452 R	12	12	100.00	24	24	100.00
501 N	15	15	91.67	21	21	100.00
501Y	12	12	90.48	24	24	100.00
501Y-Om	3	3	100.00	33	33	100.00
501Y-Om2	6	6	100.00	30	30	100.00
417 K	12	11	100.00	24	24	100.00
417 N	21	19	100.00	15	15	100.00
417 T	3	3	100.00	33	33	100.00
614D	3	3	100.00	33	33	100.00
614 G	32	32	100.00	3	3	100.00

Single-SNV discrimination performance of 36 runs including SARS-CoV-2 Alpha, Beta, Gamma, Delta, Omicron, and Kappa variants. Specificity is the percentage of runs in which the probe was correctly considered non-detecting. Sensitivity is the percentage of runs in which the probe was correctly considered detecting. Note that the 614 probe was obscured in one run and was not counted.

Together, these data highlight the ability of robust probes and SNV-discriminatory probes to work in tandem to quickly identify the RNA target present (with the robust probe) and provide single-nucleotide resolution (with the SNV-discriminatory probe).

### Evaluating the limit of detection of RT-caPCR

We next performed a series of experiments to determine the limit of detection of our RT-caPCR assay. In all previous tests in Fig. 4, a viral input of 10^7^ molecules was spiked into the VTM prior to extraction, similar to estimates of peak viral load for Omicron and Delta patients^15,16^. Here we quantified the LOD of our assay by varying the number of input copies of viral RNA and keeping the control copies the same. Using purified RNA either obtained from Twist Bioscience or synthesized in-house from gene fragments via IVT, we achieved a 1,000-molecule LOD for all 7 coronaviruses. Representative results are shown in Fig. S3. In both Fig. S3a and S3b, we find that we can successfully detect down to 1,000 molecules per reaction for a single target with both controls (HCoV 229E; Fig. S3a) and for a SARS-CoV-2 variant with both controls (Omicron; Fig. S3b), a quantity 3 orders of magnitude below that used for VTM testing when using purified RNA. When only the presence of a single target is varied, as in Fig. S3a, we find a clear delay in threshold time for each order of magnitude for only this target, whereas the 2 controls are consistent across runs. This trend is less clear for SARS-CoV-2, which is to be expected given that this target requires simultaneous amplification of 4 amplicons and 2 controls. Competition for enzymatic resources and dNTPs appears to disrupt the clear trend observed for single-amplicon targets. In Fig. S3c, a dilution series was performed by varying the amount of input RNA to the extraction pipeline for the Omicron variant. Here, too, we see detection down to the lowest tested value (10,000-molecule input into the RNA extraction step with roughly 1,000 molecules going into the RT step due to losses during extraction and not using the full 25 µL elution volume in the reaction) and an expected delay in amplification as a function of input concentration.

### Detection of multiple targets in a single sample

Having demonstrated the success of our tunable-probe RT-caPCR assay at identifying RNA targets with robust probes (each virus) with single nucleotide specificity with SNV-discriminating probes (SARS-CoV-2 variants), we next explored how our assay performed when assessing more complex inputs by simulating co-infection samples containing multiple RNA targets. Here, an input amount of 10^5^ molecules of each template was spiked directly into the RT-caPCR reaction with no VTM extraction steps. We also expanded our RT-caPCR assay to test for more RNA targets by including 3 additional primer sets and 4 probes to test for influenza A and B (2 targeting the M1 matrix protein gene of influenza A^17^ and 2 targeting nonstructural protein-1 (nsp1) in the Yamagata and Victoria lineages of influenza B^18^). This 14-plex assay was tested for all 4 new targets to confirm its efficacy (Fig. S4).

**Fig. 5:**
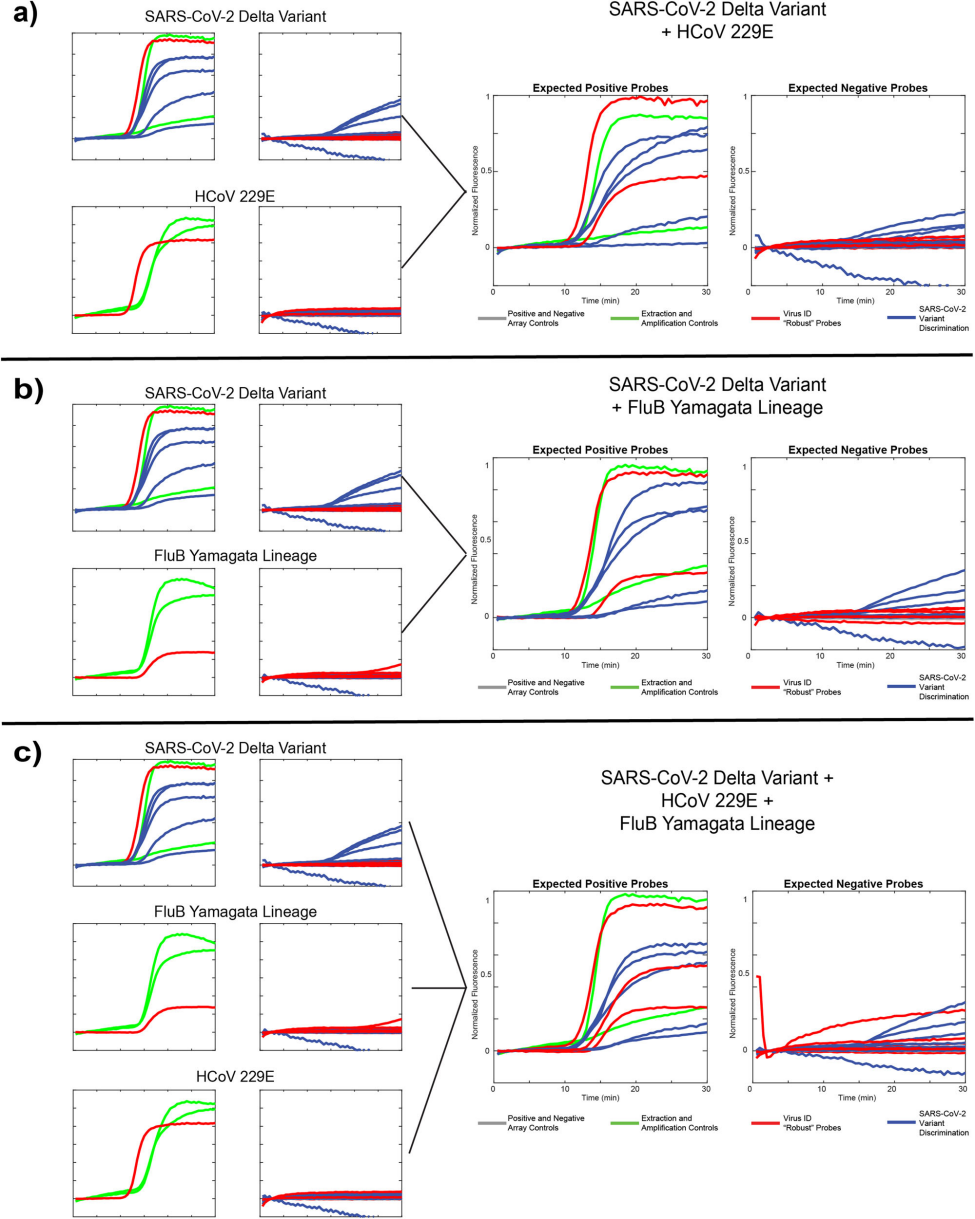
RT-caPCR featuring both robust and SNV-discriminatory probes accurately identify samples containing multiple targets. **a** Single-template results from the SARS-CoV-2 Delta variant and human coronavirus 229E along with a “coinfection” sample including both templates. **b** Single-template results from the SARS-CoV-2 Delta variant and the Yamagata lineage of influenza B, along with a coinfection sample including both templates. **c**. Single-target results from the same targets as in **a** and **b**, along with a coinfection sample including all three templates.

We assessed each of 3 different viral templates alone as well as in 3 different combinations: SARS-CoV-2 Delta variant + HCoV229E (Fig. 5a), SARS-CoV-2 Delta variant + FluB Yamagata lineage (Fig. 5b), and SARS-CoV-2 + HCoV229E + FluB Yamagata lineage (Fig. 5c). Data is presented as compressed fluorescent trace images as described above with expected positive probes in the left panel and expected negative probes in the right panel. Across all runs, we observed the expected behavior: robust (virus identification) probes report the presence of all pathogen templates included, and SNV-discriminating probes (variant identification) correctly identify the Delta variant with low levels of off-target probe activity across all 5 SNV sites. The 484E probe is the weakest performer, likely owing to the aforementioned mismatch in the domain region of this probe. Additionally, in some experiments, the MS2 extraction control performed poorly owing to competition for resources amongst 8 different amplicons. We also note that these runs only include 1 replicate of each the MS2 and IPC controls, rather than 2 as in Fig. 4, as this was an updated panel including the 501Y-Om2 probe. These data demonstrate the ability of our technology to accurately assess more complex samples containing multiple RNA targets of interest, showcasing the versatility of the assay.

## Discussion

The work presented here highlights the performance of our newly developed RT-caPCR technology featuring tunable TMSD probes with programmable sequence selectivity. The workflow permits highly multiplexed single-nucleotide resolution previously only achievable via RNAseq while improving upon the turn-around time of a traditional RT-qPCR assay, resulting in a one-pot RT-caPCR assay that is unique in both speed and depth of information provided (Fig. 2). For an assay to have high clinical sensitivity, it is important that new strains can still be detected even as mutations arise. With robust probes, we can detect all target sequences with up to a 2-nucleotide difference from the wildtype sequence, thus future-proofing our assay given that in an evolution cycle, it is unlikely that more than 3 new mutations would arise in a single 50-70 nucleotide target (the length of our TMSD probe). As such, the assay will remain functional while continued pathogen sequencing surveillance informs the design of new probes to add to the array to keep it up-to-date with emerging pathogen strains. RT-caPCR also allows for both a much faster turn-around time than traditional RT-PCR (within 40 minutes from purified RNA to answer) as well as an increase in the number of targets surveilled. Preliminary experiments decreasing the length of the reverse transcription stage suggest that this may further reduce the assay runtime, as a 5-minute RT stage performs similarly to a 10-minute one (Fig. S5). Increasing target surveillance contributes to the functionality of an assay especially when diagnosing individuals presenting with non-specific symptoms: here, we show how increasing the multiplexing capability of a test for respiratory viruses can improve healthcare outcomes by providing a more thorough examination of potential causes for acute illness from a single sample. Taken together, we anticipate that the increase in clinical sensitivity, improved turn-around time, and increase in multiplexing capability will result in a clinical impact of more thorough and effective surveillance of emerging pathogen strains.

Fine-tuning probe energetics revealed a spectrum of probe designs that allow the researcher to choose the outcome most well-suited to their assay, ranging from robust probes that will detect a target sequence with up to 2 mutations to SNV-discriminatory probes that require an exact sequence match for detection (Fig. 3). This proof-of-concept study in exploring the functionality of the RT-caPCR assay examined the real-life problem of respiratory virus diagnostics that came to light during the SARS-CoV-2 pandemic, where, to differentiate between similar disease presentations, both sensitivity in detection of pathogen and single-nucleotide resolution are required to identify the infecting virus and the infecting variant. Across our panel of respiratory viruses, we achieved excellent sensitivity in detecting true positive samples and specificity in detecting true negatives using our robust probes (Table 1) and SNV-discriminatory probes (Table 2). This capability for highly programmable sequence selectivity in probe design demonstrates the strength of our technology for the wide range of potential applications beyond diagnostics into the laboratory research space.

The innovations in robustness and target sequence specificity in probe design presented here demonstrate the positive impact RT-caPCR can have on the clinical sensitivity and epidemiological tracking for applications such as respiratory virus diagnostics. However, the implications of RT-caPCR goes far beyond the scope of diagnostics: for example, we anticipate this technology finding utility in the RNAseq space for applications such as transcriptomics due to its capacity to examine many targets while retaining single-nucleotide resolution. Additionally, our tunable probe design can be used as a standalone tool for DNA detection on the caPCR platform for research applications such as phylogeny tracking. Together, the components of this RT-caPCR assay present a viable future alternative to traditional RT-qPCR as well as RNAseq analysis by allowing for single-nucleotide resolution, improving upon turn-around time, and decreasing labor with a broad range of anticipated implementations.

## Materials and methods

### General considerations on thermodynamics of toehold probes

During the probe design, we varied the toehold binding energy for a fully complementary sequence according to the target energetic scheme. Toehold binding with variant amplicon sequence would have higher (i.e., less negative) energy, and thus would be less favorable for toehold displacement reaction initiation, which in turn would result in no or weak fluorescence signal increase. The arm strand bound to the anchor strand (attached to the surface) with binding energy of around −24 kcal mol^−1^. Presented probe energetics were calculated for 60 °C and concentration of Mg^2+^ cations at 6 mM, using a MATLAB script written in-house.

### DNA oligonucleotides

All DNA oligonucleotides used in the study were purchased from Integrated DNA Technology (IDT) or Sigma, except for the strands labeled with the BHQ-2 moiety, which were purchased from Biosearch Technologies. All PCR primers were ordered as standard desalted oligonucleotides. All fluorophore-labeled oligonucleotides, quencher-labeled oligonucleotides and unlabeled oligonucleotides shorter than 50 nt were ordered with reverse-phase HPLC purification. All unlabeled oligonucleotides longer than 50 nt were ordered with PAGE purification and were resuspended in TE Buffer pH 8.0 (IDT).

### Templates for robust probe design

DNA sequences for robust probe energetic tests were designed based on the SARS-CoV-2 Wuhan-Hu-1 Spike protein gene sequence (accession EPI_ISL_710528). Each template sequence comprised the 500 bp upstream and downstream of the 484-mutation site flanked by a randomly generated sequence between 100 and 120 bp at both the 5’ and 3’ ends. 4 templates were ordered corresponding to the 4 different nucleotides possible at position 1450 in the SARS-CoV-2 spike protein. These yield codons for 484E (GAA), 484 K (AAA), 484Q (CAA), and 484STOP (TAA)^9^. To test the ability of the probes to detect templates in the presence of mutations elsewhere in the probe sequence, variants of each of these 4 templates were constructed by introducing 1 or 2 further mutations found in other SARS-CoV-2 lineages. The 1-mutation variant included the T478K mutation (ACA → AAA) found in both the Delta AY and Omicron lineages, and the 2-mutation variant also included the S477N mutation (AGC → AAC) found in Omicron lineages^10^. Templates were ordered as sequences inserted into pUC57 plasmids from GenScript. Probes were designed using a custom MATLAB script to match each of the 4 ‘variants’ according to each of the 6 energetic criteria specified in Fig. 3. Energetics were calculated according to the SantaLucia nearest-neighbor model^19^. See Table S6 for full probe sequence information.

Templates for testing robust SARS-CoV-1/2 discrimination were designed based on the same accession specified above for SARS-CoV-2 as well as NC_004718.3 for SARS-CoV-1. On-target SARS-CoV-2 and SARS-CoV-1 sequences were based on their respective genomes, comprising 500 bp in either direction from the center of our SARS-CoV-1/2 probe sequence (nucleotide position 344 of the SARS-CoV-2 nucleocapsid). As above, these sequences were flanked by randomly generated sequences between 100 and 120 bp at both the 5’ and 3’ ends and were inserted into pUC57 plasmids. See Table S7 and S8 for 484 and SARS-CoV template sequences, respectively.

All robust probe tests were performed using a ‘standard’ master mix lacking the reverse transcriptase and RNase inhibitor found in the RT master mix for 30 minutes on donut PCR chips without the RT stage. All reactions were performed using an input copy number of 100,000 molecules, determined based on spectrophotometer measurements and the molecular weight of the plasmid with the inserted sequence. All tests were performed using the full 14-plex primer composition.

### Templates for RNA viruses

Templates for all SARS-CoV-2 variants (Wuhan,Two Alpha variants, Beta, Delta, Delta AY.1, Delta AY.2, Gamma, Kappa, Omicron BA.1, and 2 Omicron BA.2 variants), human coronaviruses 229E and NL63, influenza viruses H1N1 and H3N2 (Influenza A) and Yamagata lineage (Influenza B) were purchased from Twist Bioscience at a concentration of 10^6^ molecules/μL (See Table S5 for product identifiers). Templates for SARS-CoV-1, human coronaviruses HKU1 and OC43, MERS-CoV, influenza B Victoria lineage, and the internal control IPC-1 were synthesized via in vitro transcription (IVT) as described in Supplemental Methods. MS2 RNA was purchased from Millipore Sigma at 10^11^ molecules/μL.

### Amplicon design for viral identification

The nucleocapsid gene of SARS-CoV-2 was selected for universal SARS-CoV-2 detection, given its relatively more conserved sequence and common use in other RT-PCR diagnostic platforms. We identified a conserved region between SARS-CoV-1 and SARS-CoV-2 in the nucleocapsid (N) gene that allows for a single set of primers to be used, but which also feature enough mutations between the 2 viruses to discriminate using toehold probes.

We used the sequence of the synthetic genome purchased from Twist Bioscience and identified regions within the *orf1ab* gene for each of 4 other human coronavirus targets, 229E, NL63, HKU1, and OC43. The MERS-CoV amplicon region was designed by examining >700 MERS-CoV sequences from the National Center for Biotechnology Information (NCBI)’s Genome browser, aligning them in Geneious Prime, and identifying a conserved region. A similar methodology was used to design amplicons targeting the *M1* matrix protein of influenza A and nonstructural protein-1 (*nsp1*) in the Yamagata and Victoria lineages of influenza B. Probe sequences for viral identification and SARS-CoV-2 variant discrimination are listed in Table S9.

### Amplicon design for SNV discrimination

Amplicon regions for SNV-specific mutations in the SARS-CoV-2 spike protein were designed to capture as many commonly mutated residues as can fit in approximately 200 bp. This resulted in 2 amplicons containing 2 mutation sites each (K417 and L452 in one; E484 and N501 in the other), and a separate amplicon for D614^9^. Probes were targeted such that the toehold region is centered on the SNV.

### Preparation of contrived clinical samples

VTM samples testing negative for presence of SARS-CoV-2 were purchased from Discovery Life Sciences’ sample repository. Sample lysis and nucleic acid purification was performed using standard magnetic bead-based purification protocols^13^. 100 μL of VTM was added to 500 μL 6 M guanidine thiocyanate (Sigma Aldrich) prepared in 1X TE pH 6.5 buffer, 20 μL proteinase K (Qiagen), 6 μL 10% Triton X-100, 24.8 μL 1 M DTT, and 1 μL of 1 μg/μL carrier RNA. Heat treatment was performed by incubation at 56 °C for 5 minutes, after which the sample was spiked with 10 μL of 10^6^ copies/μL synthetic target RNA (from either Twist Bioscience or prepared through in vitro transcription of gene fragments) and 10 μL MS2 external extraction control RNA (Millipore Sigma) diluted to 10^5^ molecules/μL.

### Extraction and purification of RNA

A magnetic bead-based extraction method was used for all contrived samples^13^. Extraction was performed by spiking 15 μL of MagneSil paramagnetic beads (Promega) into the mixture and incubating at room temperature for 5 minutes with intermittent tube inversion to maintain suspension. Magnetic beads were collected on a magnetic stand rack and supernatant was removed from the beads. The beads were washed twice with 600 μL 70% ethanol and air-dried for 10 minutes at room temperature. RNA was eluted in 25 μL nuclease-free water. After a 2-minute incubation, beads were separated on a magnetic rack and the supernatant was transferred to a clean 0.6 mL tube (Genesee Scientific) and stored at -80 °C until use.

### Primer design

Following selection of an amplicon region, primer design was performed using the Simulated Annealing Design using Dimer Likelihood Estimation (SADDLE) algorithm^20^. SARS-CoV-2-related primers, including the SARS-CoV-1/2 discriminatory set, were designed first, given the relatively limited available region for primer placement, and the remaining 5 coronaviruses, along with the MS2 and internal positive control (IPC) controls, were subsequently designed using the other 4 primer sets as existing sequences. Influenza primers were designed last. Main considerations during primer design were as follows: 1) amplicon length was restricted to 250 bp or shorter; 2) standard free energy of primer binding (ΔG°) was in the range of −11 to −12.5 kcal mol^−1^ at 60 °C in a solution with 6 mM Mg^2+^ concentration. See Table S10 for the primer sequences.

### RT-caPCR reaction

Each toroidal RT-caPCR reaction was performed with 20 units of AptaTaq Δexo DNA Polymerase (Roche Custom Biotech) and 100 units of NxtScript reverse transcriptase (Roche Custom Biotech) in 1× AptaTaq Fast PCR buffer supplemented with MgCl_2_ for a final MgCl_2_ concentration of 6 mM per reaction. Each PCR reaction mixture was supplied with 10 units of RNaseOUT ribonuclease inhibitor (Invitrogen) to limit RNase-mediated degradation. Each reaction includes 0.25 mM concentration of each of the standard deoxynucleotide triphosphates (dATP, dTTP, dGTP, dCTP), approximately 5,000 molecules of IVT-synthesized IPC, and all appropriate primers. While we had initially been using Asuragen’s Armored IPC RNA, we moved away from this to limit the uncertainty associated with the thermal release protocol and prepared IVT-synthesized IPC RNA with the same sequence. This template was found to perform similarly (Fig. S6). Primer concentrations varied from 250 to 1500 nM depending on the target due to variable PCR efficiency across different primer pairs; concentrations that were used equalize their performance in a single reaction. Asymmetric primer concentrations were used such that the primer used for cDNA synthesis is the same primer used to synthesize the amplicon strand which displaces the quencher on the toehold probes. A 1:2 ratio of forward primer:reverse primer was used for all targets.

Reverse transcription was performed with all toroidal RT-caPCR instrument heaters set to 42 °C for 10 minutes, after which heaters were set to 57 °C (array side) and 95 °C for 30 minutes. Images were acquired every 30 seconds during the PCR phase only.

### Data analysis

Raw images were processed using custom MATLAB functions and scripts. Masks for each probe array spot were constructed to extract raw intensity of each probe at every time point, after which intensity of the background and of the spot-specific baseline were subtracted from the intensity at each time point. All spots are normalized to the brightest fluorescence value across every spot on the probe array. SNV-specific probes for SARS-CoV-2 variant identification are treated by considering all probes associated with a single mutation site of interest and identifying the probe that exhibits the maximum slope of the fluorescence curve. See Supplemental Methods for an in-depth description of the full analysis pipeline.

### Statistics and reproducibility

No statistical calculations were performed for the presented data. Samples sizes are given in Tables 1 and 2 for all contrived sample runs that were valid according to the criteria outlined in the text. Experiments presented in Figs. 3 and 5 were preformed once for each condition.

### Reporting summary

Further information on research design is available in the Nature Portfolio Reporting Summary linked to this article.

### Supplementary information

Supplemental Materials

Reporting Summary

## Data Availability

The main data supporting the results in this study are available within the paper and its Supplementary Information. The raw and analyzed fluorescent value datasets that support the findings of this study are available on figshare with the identifier 10.6084/m9.figshare.24069234.v1^21^. Some exemplary raw data are also available at https://github.com/tr-sullivan/coronavirus-analysis.

## Code availability

The MATLAB code used for fluorescent image analysis and post processing is available at https://github.com/tr-sullivan/coronavirus-analysis^22^. MATLAB version R2020b was used.
