# Supplementary material for: Rapid, tunable, and multiplexed detection of RNA using convective array PCR
Source: Commun Biol. 2023 Sep 23;6:973. doi: 10.1038/s42003-023-05346-4 (PMC10518007; doi:10.1038/s42003-023-05346-4)
Supplement: Supplementary file 1 — Supplemental Materials [file 42003_2023_5346_MOESM1_ESM.pdf]

Supplemental Methods:

***In Vitro* Transcription for RNA targets.** For targets not available in Twist Bioscience's respiratory viral controls catalog, gBlocks gene fragments were ordered from Integrated DNA Technologies (IDT) containing an approximately 1 kb sequence including the ~200 bp amplicon sequence of interest, flanked by a T7 transcription promoter (TAATACGACTCACTATAGGG) sequence and a T7 terminator sequence (CTAGCATAACCCCTTGGGGCCTCTAAACGGGTCTTGAGGGGTTTTTTG) upstream and downstream of the amplicon, respectively. This region was further flanked by M13 primer binding sites (GTAAAACGACGGCCAGT and GTCATAGCTGTTTCCTG) for amplification of the received template. Short random sequences were inserted between the amplicon and promoter/terminator sequences as well as at the 5' and 3' ends of the fragment. gBlocks were amplified using Phusion Hot-Start Flex 2X Master Mix (New England Biolabs), 500 nM M13 forward and reverse primers (IDT), ~10^8 copies of template, and 3% DMSO. Amplification was performed following NEB's Phusion protocol on an Eppendorf Mastercycler nexus thermocycler. Annealing temperature was estimated using NEB's melting temperature (Tm) calculator. After amplification, samples were purified using Agencourt AMPure XP SPRI beads following the recommended protocol, including bead binding, two ethanol washes, drying, and elution in 40 µL of DEPC-treated water. Samples were run on E-Gel agarose gels (Invitrogen) for 30 minutes in an E-Gel Simple Runner, and fluorescence intensity relative to the GeneRuler 100 bp Plus DNA ladder (Thermo Scientific) was used to estimate concentration of the product (mass of 3000 bp band = 56 ng).

IVT was performed using a MEGAScript T7 Transcription Kit (Invitrogen), including 1 µL of purified PCR product (~500 ng), 2 µL each of ATP, GTP, CTP, and UTP stocks, 2 µL of 10X reaction buffer, and water up to 20 µL. Samples were incubated on a heat block at 37 °C overnight for >12 hours. RNA was purified by adding 1 µL TURBO DNase (Invitrogen) and incubating at 37 °C for 30 minutes, followed by lithium chloride precipitation (30 µL LiCl

precipitation solution + 30 µL nuclease-free water) including 30-minute precipitation at -20 °C, centrifugation for 15 minutes at °C, ethanol wash, drying, and elution in 100 µL nuclease-free water. Concentrations of final RNA stocks were quantified using a DeNovix DS-11 Fluorometer/Spectrophotometer.

Surface functionalization. Attachment of the pre-annealed probe to the surface of the fluidic chamber is performed via strain-promoted azide–alkyne cycloaddition between the dibenzocyclooctyne-functionalized probe and the azide (N3)-functionalized surface of the fluidic chamber. N3 surface functionalization is performed according to the following protocol. Step 1: glass slides are treated with sodium hydroxide solution (10 M) for 30min in Branson ultrasonic bath (Bransonic CPXH Digital Bath 2800) at the high power of ultrasound generation. Step 2: After washing with copious amounts of water and drying at 120 °C, the slides are treated with oxygen plasma for 30 min in a Harrick plasma cleaner at high power and maintaining oxygen pressure in a range of 0.4–0.6 torr. Step 3: hydroxylated slides are treated with a 5% solution of N-(2-aminoethyl)-2,2,4- trimethyl-1-azasila cyclopentane (Gelest) in dry dichloromethane (Sigma) for 16 h at room temperature, followed by washing with 95% ethanol in water solution; amino-silanized slides are baked in ambient atmosphere at 105 °C for 2 h. Step 4: functionalization of the amino-terminated slides with an isothiocyanate moiety is performed with 10 mM solution of p-phenylene diisothiocyanate (Sigma) in dry pyridine-dimethylformamide (both chemicals are from Sigma) mixture (9:1, v/v) for 2 h at room temperature, followed by washing with 95% ethanol in water solution and drying at 80 °C for 1 h. Step 5: azide functionalization of the isothiocyanate slides is performed at 37 °C for 2 h with a solution of 11-azido-3,6,9-trioxaundecan-1-amine (50 µl) in 0.1 M phosphate buffer pH 8 (10 mL), followed by washing with copious amount of water and drying at 45 °C. At this stage, the slides are ready for microarray printing or can be stored vacuum-sealed at 4 °C for at least 6 months without loss of conjugation activity.

For data shown in **Figure 3** and **Figure 5,** arrays were spotted onto COP rather than glass using a broadly similar method to that described above but lacking the NaOH step and with a shortened plasma treatment (10 minutes).

Probe annealing. Toehold microarray probes were annealed in 2× PBS buffer according to the following protocol: 90 °C for 3 min, then uniformly decreasing temperature down to 30 °C over a period of 1 h. Concentrations of the probe components for the universal probe architecture were as follows: anchor strand − 2 μM, arm strand − 3 μM, quencher strand − 4.5 μM. Concentrations of the probe components for the super universal probe architecture were as follows: anchor strand − 2 μM, arm strand − 3 μM, quencher arm strand − 4.5μM, universal quencher strand − 6.75 μM.

Microarray spotting solution preparation. Microarray spotting solution was prepared by diluting the annealed probes with Tris-HCl buffer spiked with $MgCl_2$ to obtain the following final concentration of the components: 1× Tris-HCl buffer (20 mM Tris, 50 mM KCl), 1.5 mM $MgCl_2$, 0.5 μM of the probe, recalculated to the concentration of the anchor strand.

Microarray printing. Microarrays were printed using a Scienion sciFLEXARRAYER S3 microarray printer equipped with PDC 60 microcapillaries. 70% relative humidity was maintained in the printing enclosure during the printing process. After printing the arrays remain in the humidity controlled printing enclosure for 20 minutes, then are stored in a desiccator under reduced pressure for a minimum of 30 minutes to dry. Microarrays were stored at room temperature in a desiccator until final assembly and use.

Extended Data Analysis Description.

Intensity Extraction. All data treatment and visualization was executed in MATLAB. After rescaling the raw intensity of all images, semi-automated masking was performed using the *imfindcircles* MATLAB function based on a circular Hough transform. This yields a binary matrix for each spot specifying which pixels are included in each region-of-interest (ROI). These masks

were used to extract the average intensity of each probe location at each time point, $f_i(t)\ \forall\ i\ \in\ 1,2,\ldots,n_{spot}$.

Background and Baseline Subtraction. For each ROI, we constructed a local annular background ROI by extending the microarray spot size by 10 pixels in all directions and removing the microarray ROI. The intensity of this background region, $bg_i(t)$, was obtained for each image and was subtracted from the raw fluorescence for the corresponding spot to remove the influence of fluctuations in illumination intensity and ambient lighting.

$$\hat{f}_i(t) = f_i(t) - bg_i(t)$$

The starting intensity of each microarray spot is brighter than the background and varies depending on the probe and the microarray print batch. To remove the influence of this factor, we calculated a 'baseline' intensity for each spot as the average intensity of the spot from images 3 to 7 (i.e. from 1.5 to 3.5 minutes after beginning the run). The baseline does not include the first minute of the run as probes typically alter their starting fluorescence during thermal equilibration and flow stabilization.

$f_i^*(t) = \hat{f}_i(t) - f_{i,baseline}$ where

$$f_{i,baseline} = mean(\hat{f}_i(t))\ \forall\ t\ \in 1.5,\ldots,3.5$$

Normalization. All spots on the array are normalized to the brightest fluorescence value found anywhere within the fluorescence data set. Positive control spots (the four corners as well as spot (2,3)) are normalized in the same way but then set to a starting value of 1.

$$f_{i.norm}(t) = f_i^*(t)\ /\ m \text{ where}$$

$$m = max(max(f_i^*(t))\ \ i\ \in\ 1,2,\ldots,n_{spot})$$

Threshold time (T_t) values for each detecting probe are calculated by identifying the last spot below 15% of the final fluorescence and the first spot above and performing a linear interpolation between the two values.

SNV Identification. Each of the five different SARS-CoV-2 mutation sites (417, 452, 484, 501, and 614) are considered separately. For a given site, the gradient is estimated at all time points beyond the end of the baseline using a fourth-order central difference approximation (note that the ending time point is 29 minutes for this calculation owing to the need for two later images):

$$f'_i(t) = \frac{-f_i^*(t+1) + 8f_i^*(t+0.5) - 8f_i^*(t-0.5) + f_i^*(t-1)}{12 * 0.5} \forall t \in 4, \ldots, 29$$

We have found that using a higher-order estimate for the derivative such as this one allows for more accurate SNV calling by limiting the influence of small fluctuations that may be caused by dust particles circulating through the ROI or other local changes that are not removed by our data treatment pipeline. Simpler estimates, such as using the difference in intensity between successive images, are less robust.

Once the slope is estimated for each probe associated with the mutation site in question, the probe with the maximum slope at any point between 4 minutes and 29 minutes is selected as the dominant SNV. As above, all probes associated with this mutation site are normalized as to the maximum fluorescence value across the entire microarray.

Supplemental Tables

Age	Min	Max	Mean	Median	Standard Deviation
Value	0.08	89	52	60	25.5

Table S1

Ethnicity	Non-Hispanic/Latino	Unknown
Count	47	3

Table S2

Race	White	Black	American Indian	Asian	Unknown
Count	40	5	1	1	3

Table S3

Sex	Male	Female
Count	25	25

Table S4

Table S1-S4. VTM Statistics. Summary information for all VTM samples used for extraction testing. Age is given in years. All other quantities are given as the number falling into each category (out of 50 total VTMs).

Target	Source of Template	Product ID No. (If Applicable)	Accession ID for Sequence
SARS-CoV-2 Wuhan	Twist Bioscience	Control 2	MN908947.3
SARS-CoV-2 Alpha 1	Twist Bioscience	Control 14	EPI_ISL_710528
SARS-CoV-2 Alpha 2	Twist Bioscience	Control 15	EPI_ISL_601443
SARS-CoV-2 Beta	Twist Bioscience	Control 16	EPI_ISL_678597
SARS-CoV-2 Gamma	Twist Bioscience	Control 17	EPI_ISL_792683
SARS-CoV-2 Delta	Twist Bioscience	Control 23	EPI_ISL_1544014
SARS-CoV-2 Delta AY.1	Twist Bioscience	Control 28	EPI_ISL_2695467
SARS-CoV-2 Delta AY.2	Twist Bioscience	Control 29	EPI_ISL_2693246
SARS-CoV-2 Kappa	Twist Bioscience	Control 18	EPI_ISL_1662307
SARS-CoV-2 Omicron BA1	Twist Bioscience	Control 48	EPI_ISL_6841980
SARS-CoV-2 Omicron BA2.1	Twist Bioscience	Control 50	EPI_ISL_7190366
SARS-CoV-2 Omicron BA2.2	Twist Bioscience	Control 51	EPI_ISL_7718520
SARS-CoV-1	IVT	N/A	NC_004718.3
MERS-CoV	IVT	N/A	NC_019843.3
HCoV 229E	Twist Bioscience	N/A	NC_002645.1
HCoV NL63	Twist Bioscience	N/A	NC_005831.2
HCoV OC43	IVT	N/A	NC_006213.1
HCoV HKU1	IVT	N/A	NC_006577.2

Table S5. Panel Product IDs. Product information used for each template source. All SARS-CoV-2 templates and human coronaviruses 229E and NL63 were obtained from Twist Bioscience, according to the Control number identifier given in column 3. All other templates

were synthesized via IVT. The final column gives the reference accession number for each template. These sequences were used for primer and probe design.

Probe	Sequence
484E_1	ggagtgggtcAAATCTATCAGGCCGGTAGCACACCTTGTAATGGTGTT**G**AAGGTTTTAAT
484K_1	ctttctccgcTGAAATCTATCAGGCCGGTAGCACACCTTGTAATGGTGTT**A**AAGGTTTTAATT
484Q_1	ggagtgggtttAATCTATCAGGCCGGTAGCACACCTTGTAATGGTGTT**C**AAGGTTTTAAT
484STOP_1	ctgaatacaccgAAATCTATCAGGCCGGTAGCACACCTTGTAATGGTGTT**T**AAGGTTTTAATT
484E_2	gggagtggTGAAATCTATCAGGCCGGTAGCACACCTTGTAATGGTGTT**G**AAGGTTTTAAT
484K_2	gctttctccCTGAAATCTATCAGGCCGGTAGCACACCTTGTAATGGTGTT**A**AAGGTTTTAATT
484Q_2	gggagtggTGAAATCTATCAGGCCGGTAGCACACCTTGTAATGGTGTT**C**AAGGTTTTAAT
484STOP_2	gctgaatacCTGAAATCTATCAGGCCGGTAGCACACCTTGTAATGGTGTT**T**AAGGTTTTAATT
484E_3	ccaaaagTTTCAACTGAAATCTATCAGGCCGGTAGCACACCTTGTAATGGTGTT**G**AAGGTTTTAAT
484K_3	ggcaATATTTCAACTGAAATCTATCAGGCCGGTAGCACACCTTGTAATGGTGTT**A**AAGGTTTTAATT
484Q_3	gcgTTTCAACTGAAATCTATCAGGCCGGTAGCACACCTTGTAATGGTGTT**C**AAGGTTTTAAT
484STOP_3	agctTTTCAACTGAAATCTATCAGGCCGGTAGCACACCTTGTAATGGTGTT**T**AAGGTTTTAATT
484E_4	GAGAGATATTTCAACTGAAATCTATCAGGCCGGTAGCACACCTTGTAATGGTGTT**G**AAGGTTTTAAT
484K_4	AGAGAGATATTTCAACTGAAATCTATCAGGCCGGTAGCACACCTTGTAATGGTGTT**A**AAGGTTTTAATT
484Q_4	GAGAGATATTTCAACTGAAATCTATCAGGCCGGTAGCACACCTTGTAATGGTGTT**C**AAGGTTTTAAT
484STOP_4	AGAGATATTTCAACTGAAATCTATCAGGCCGGTAGCACACCTTGTAATGGTGTT**T**AAGGTTTTAATT
484E_5	GAGAGATATTTCAACTGAAATCTATCAGGCCGGTAGCACACCTTGTAATGGTGTT**G**AAGGTTTTAAT
484K_5	AGAGAGATATTTCAACTGAAATCTATCAGGCCGGTAGCACACCTTGTAATGGTGTT**A**AAGGTTTTAATT
484Q_5	GAGAGATATTTCAACTGAAATCTATCAGGCCGGTAGCACACCTTGTAATGGTGTT**C**AAGGTTTTAAT
484STOP_5	AGAGATATTTCAACTGAAATCTATCAGGCCGGTAGCACACCTTGTAATGGTGTTT**A**AGGTTTTAATT
484E_6	GAGAGATATTTCAACTGAAATCTATCAGGCCGGTAGCACACCTTGTAATGGTGTT**G**AAGGTTTTAAT
484K_6	AGAGAGATATTTCAACTGAAATCTATCAGGCCGGTAGCACACCTTGTAATGGTGTT**A**AAGGTTTTAATT
484Q_6	GAGAGATATTTCAACTGAAATCTATCAGGCCGGTAGCACACCTTGTAATGGTGTT**C**AAGGTTTTAAT
484STOP_6	AGAGATATTTCAACTGAAATCTATCAGGCCGGTAGCACACCTTGTAATGGTGTT**T**AAGGTTTTAATT

Table S6. 484 Probe Sequences. Sequences (5’ → 3’) used for optimizing probe energetics along the sensitivity-specificity axis. Non-homologous regions (NHRs) are shown in pink and lowercase and are only present in the first three energetic schemes. The double-stranded domain region is in black. The single-stranded toehold region is colored according to the scheme used in **Figure 3** (blue for 484E, red for 484K, green for 484Q, gray for 484STOP). The SNV difference between the four variants is in bold.

Template	Sequence
484E	GACCTTGCTTAACGCTAGCTTGGTAACGCGAGCACTCCGTTGCGGCGTAAAACGACGGCCAGTTAATACGACTCACTATAGGGTACTGGAGGCGACGGGTAGGACTTTAGAGTCCAACCAACAGAATCTATTGTTAGA<br>TTTCCTAATATTACAAACTTGTGCCCTTTTGGTGAAGTTTTAACGCCACCAGATTTGCATCTGTTTATGCTTGGAACAGGAAGAGAATCAGCAACTGTGTTGCTGATTATTCTGTCCTATATAATTCCGCATCATTTTCCA<br>CTTTTAAGTGTTATGGAGTGTCTCCTACTAAATTAAATGATCTCTGCTTTACTAATGTCTATGCAGATTCATTTGTAATTAGAGGTGATGAAGTCAGACAAATCGCTCCAGGGCAAACTGGAAAGATTGCTGATTATAAT<br>TATAAATTACCAGATGATTTTACAGGCTGCGTTATAGCTTGGAATTCTAACAATCTTGATTCTAAGGTTGGTGGTAATTATAATTACCTGTATAGATTGTTTAGGAAGTCTAATCTCAAACCTTTTGA**GAGAGATATTTCA**<br>**ACTGAAATCTATCAGGCCGGTAGCACACCTTGTAATGGTGTTGAAGGTTTTAAT**TGTTACTTTCCTTTACAATCATATGGTTTCCAACCCACTAATGGTGTTGGTTACCAACCATACAGAGTAGTAGTACTTTCTTTTGAA<br>CTTCTACATGCACCAGCAACTGTTTGTGGACCTAAAAAGTCTACTAATTTGGTTAAAAACAAATGTGTCAATTTCAACTTCAATGGTTTAACAGGCACAGGTGTTCTTACTGAGTCTAACAAAAAGTTTCTGCCTTTCCAA<br>CAATTTGGCAGAGACATTGCTGACACTACTGATGCTGTCCGTGATCCACAGACACTTGAGATTCTTGACATTACACCATGTTCTTTTGGTGGTGTCAGTGTTATAACACCAGGAACAAATACTTCTAACCAGGTTGCTGT<br>TCTTTATCAGGATGTTAACTGCACAGAAGTCCCTGTTGCTATTCATGCAGATCAACTTACTCCTACTTGGCGTGTTTATTCTACAGGTTCTAATGTTTTTCAAACACGTGCAGGCTGTTTAGTCTATACAAGTCGACCCTA<br>GCATAACCCCTTGGGGCCTCTAAACGGGTCTTGAGGGGTTTTTTGGTCATAGCTGTTTCCTGAATGGATCACAGCTATCTTTCAAGAGTGACAGAGGGACCGCG
484K	CAGCACAAGGTAACGAAATTTCTCTATACTGAGGCCCCTTGGTCGTCGGGTAAAACGACGGCCAGTTAATACGACTCACTATAGGGCAAGTTAGTCTCACTTTAGAGTCCAACCAACAGAATCTATTGTTAGATTTCCT<br>AATATTACAAACTTGTGCCCTTTTGGTGAAGTTTTAACGCCACCAGATTTGCATCTGTTTATGCTTGGAACAGGAAGAGAATCAGCAACTGTGTTGCTGATTATTCTGTCCTATATAATTCCGCATCATTTTCCACTTTA<br>AGTGTTATGGAGTGTCTCCTACTAAATTAAATGATCTCTGCTTTACTAATGTCTATGCAGATTCATTTGTAATTAGAGGTGATGAAGTCAGACAAATCGCTCCAGGGCAAACTGGAAAGATTGCTGATTATAATTATAAA<br>TTACCAGATGATTTTACAGGCTGCGTTATAGCTTGGAATTCTAACAATCTTGATTCTAAGGTTGGTGGTAATTATAATTACCTGTATAGATTGTTTAGGAAGTCTAATCTCAAACCTTTTG**AGAGAGATATTTCAACTGAA**<br>**ATCTATCAGGCCGGTAGCACACCTTGTAATGGTGTTAAAGGTTTTAATT**GTTACTTTCCTTTACAATCATATGGTTTCCAACCCACTAATGGTGTTGGTTACCAACCATACAGAGTAGTAGTACTTTCTTTTGAACTTCTAC<br>ATGCACCAGCAACTGTTTGTGGACCTAAAAAGTCTACTAATTTGGTTAAAAACAAATGTGTCAATTTCAACTTCAATGGTTTAACAGGCACAGGTGTTCTTACTGAGTCTAACAAAAAGTTTCTGCCTTTCCAACAATTT<br>GGCAGAGACATTGCTGACACTACTGATGCTGTCCGTGATCCACAGACACTTGAGATTCTTGACATTACACCATGTTCTTTTGGTGGTGTCAGTGTTATAACACCAGGAACAAATACTTCTAACCAGGTTGCTGTTCTTTA<br>TCAGGATGTTAACTGCACAGAAGTCCCTGTTGCTATTCATGCAGATCAACTTACTCCTACTTGGCGTGTTTATTCTACAGGTTCTAATGTTTTTCAAACACGTGCAGGCTGTTTAAAAACAGGCCCCTAGCATAACCCCTT<br>GGGGCCTCTAAACGGGTCTTGAGGGGTTTTTTGGTCATAGCTGTTTCCTGTTACCAGAAAACCGCAGGCACCTCTTAACCCTACGAGTCTAC
484Q	AAGACAGTTCATAATAGGTATTGTGCCGAAACGGACTCTTCCGTAAAACGACGGCCAGTTAATACGACTCACTATAGGGTTACTTCCGAGGGACCTATAACTTTAGAGTCCAACCAACAGAATCTATTGTTAGATTTCC<br>TAATATTACAAACTTGTGCCCTTTTGGTGAAGTTTTAACGCCACCAGATTTGCATCTGTTTATGCTTGGAACAGGAAGAGAATCAGCAACTGTGTTGCTGATTATTCTGTCCTATATAATTCCGCATCATTTTCCACTTTT<br>AAGTGTTATGGAGTGTCTCCTACTAAATTAAATGATCTCTGCTTTACTAATGTCTATGCAGATTCATTTGTAATTAGAGGTGATGAAGTCAGACAAATCGCTCCAGGGCAAACTGGAAAGATTGCTGATTATAATTATAA<br>ATTACCAGATGATTTTACAGGCTGCGTTATAGCTTGGAATTCTAACAATCTTGATTCTAAGGTTGGTGGTAATTATAATTACCTGTATAGATTGTTTAGGAAGTCTAATCTCAAACCTTTTGA**GAGAGATATTTCAACTGA**<br>**AATCTATCAGGCCGGTAGCACACCTTGTAATGGTGTTCAAGGTTTTAAT**TGTTACTTTCCTTTACAATCATATGGTTTCCAACCCACTAATGGTGTTGGTTACCAACCATACAGAGTAGTAGTACTTTCTTTTGAACTTCTA<br>CATGCACCAGCAACTGTTTGTGGACCTAAAAAGTCTACTAATTTGGTTAAAAACAAATGTGTCAATTTCAACTTCAATGGTTTAACAGGCACAGGTGTTCTTACTGAGTCTAACAAAAAGTTTCTGCCTTTCCAACAATTT<br>GGCAGAGACATTGCTGACACTACTGATGCTGTCCGTGATCCACAGACACTTGAGATTCTTGACATTACACCATGTTCTTTTGGTGGTGTCAGTGTTATAACACCAGGAACAAATACTTCTAACCAGGTTGCTGTTCTTTA<br>TCAGGATGTTAACTGCACAGAAGTCCCTGTTGCTATTCATGCAGATCAACTTACTCCTACTTGGCGTGTTTATTCTACAGGTTCTAATGTTTTTCAAACACGTGCAGGCTGTTTACGTGGCTAATTCGTTGGCTAGCATAA<br>CCCCTTGGGGCCTCTAAACGGGTCTTGAGGGGTTTTTTGGTCATAGCTGTTTCCTGGAACACCGGATCCTGCGCTTTTTAAGAATGT
484STOP	GCTACCAACATCTTTAACTTCGTGTCATGCACACCTCGTGTGATCTTAGTAAAACGACGGCCAGTTAATACGACTCACTATAGGGCCGAGTCACCATACTTTAGAGTCCAACCAACAGAATCTATTGTTAGATTTCCTAA<br>TATTACAAACTTGTGCCCTTTTGGTGAAGTTTTAACGCCACCAGATTTGCATCTGTTTATGCTTGGAACAGGAAGAGAATCAGCAACTGTGTTGCTGATTATTCTGTCCTATATAATTCCGCATCATTTTCCACTTTTAAG<br>TGTTATGGAGTGTCTCCTACTAAATTAAATGATCTCTGCTTTACTAATGTCTATGCAGATTCATTTGTAATTAGAGGTGATGAAGTCAGACAAATCGCTCCAGGGCAAACTGGAAAGATTGCTGATTATAATTATAAATT<br>ACCAGATGATTTTACAGGCTGCGTTATAGCTTGGAATTCTAACAATCTTGATTCTAAGGTTGGTGGTAATTATAATTACCTGTATAGATTGTTTAGGAAGTCTAATCTCAAACCTTTTG**AGAGAGATATTTCAACTGAAA**<br>**TCTATCAGGCCGGTAGCACACCTTGTAATGGTGTTTAAGGTTTTAATT**GTTACTTTCCTTTACAATCATATGGTTTCCAACCCACTAATGGTGTTGGTTACCAACCATACAGAGTAGTAGTACTTTCTTTTGAACTTCTACA<br>TGCACCAGCAACTGTTTGTGGACCTAAAAAGTCTACTAATTTGGTTAAAAACAAATGTGTCAATTTCAACTTCAATGGTTTAACAGGCACAGGTGTTCTTACTGAGTCTAACAAAAAGTTTCTGCCTTTCCAACAATTTG<br>GCAGAGACATTGCTGACACTACTGATGCTGTCCGTGATCCACAGACACTTGAGATTCTTGACATTACACCATGTTCTTTTGGTGGTGTCAGTGTTATAACACCAGGAACAAATACTTCTAACCAGGTTGCTGTTCTTTAT<br>CAGGATGTTAACTGCACAGAAGTCCCTGTTGCTATTCATGCAGATCAACTTACTCCTACTTGGCGTGTTTATTCTACAGGTTCTAATGTTTTTCAAACACGTGCAGGCTGTTTACTCCTCGGTCTAGCTAGCATAACCCCT<br>TGGGGCCTCTAAACGGGTCTTGAGGGGTTTTTTGGTCATAGCTGTTTCCTGATGCGTATCGACCCGCGCAATCACTATAGCGACATT
484E_1mut	TTGAAAACCCGTGGGAATATTGCCTTTCATCAGACTCTCTTGTAAAACGACGGCCAGTTAATACGACTCACTATAGGGCTTTTGATAATCCTGCACTTTAGAGTCCAACCAACAGAATCTATTGTTAGATTTCCTAATATT<br>ACAAACTTGTGCCCTTTTGGTGAAGTTTTAACGCCACCAGATTTGCATCTGTTTATGCTTGGAACAGGAAGAGAATCAGCAACTGTGTTGCTGATTATTCTGTCCTATATAATTCCGCATCATTTTCCACTTTTAAGTGTT<br>ATGGAGTGTCTCCTACTAAATTAAATGATCTCTGCTTTACTAATGTCTATGCAGATTCATTTGTAATTAGAGGTGATGAAGTCAGACAAATCGCTCCAGGGCAAACTGGAAAGATTGCTGATTATAATTATAAATTACCA<br>GATGATTTTACAGGCTGCGTTATAGCTTGGAATTCTAACAATCTTGATTCTAAGGTTGGTGGTAATTATAATTACCTGTATAGATTGTTTAGGAAGTCTAATCTCAAACCTTTTGA**GAGAGATATTTCAACTGAAATCTAT**<br>**CAGGCCGGTAGCA*A*ACCTTGTAATGGTGTTGAAGGTTTTAAT**TGTTACTTTCCTTTACAATCATATGGTTTCCAACCCACTAATGGTGTTGGTTACCAACCATACAGAGTAGTAGTACTTTCTTTTGAACTTCTACATGCA<br>CCAGCAACTGTTTGTGGACCTAAAAAGTCTACTAATTTGGTTAAAAACAAATGTGTCAATTTCAACTTCAATGGTTTAACAGGCACAGGTGTTCTTACTGAGTCTAACAAAAAGTTTCTGCCTTTCCAACAATTTGGCAG<br>AGACATTGCTGACACTACTGATGCTGTCCGTGATCCACAGACACTTGAGATTCTTGACATTACACCATGTTCTTTTGGTGGTGTCAGTGTTATAACACCAGGAACAAATACTTCTAACCAGGTTGCTGTTCTTTATCAGG<br>ATGTTAACTGCACAGAAGTCCCTGTTGCTATTCATGCAGATCAACTTACTCCTACTTGGCGTGTTTATTCTACAGGTTCTAATGTTTTTCAAACACGTGCAGGCTGTTTACGGCTTTGCCAGTCTTTCTAGCATAACCCCTT<br>GGGGCCTCTAAACGGGTCTTGAGGGGTTTTTTGGTCATAGCTGTTTCCTGACCCAGCGCGCGGCAGGCTCGGAGGTGTAACTGGCTAAA
484K_1mut	GTGTAAGCGATTATCACAAAAAATCTTCTCACGGCATTCAGAAAATTAGTGTAAAACGACGGCCAGTTAATACGACTCACTATAGGGCACAAGTGTTGGGAGTGACTTTAGAGTCCAACCAACAGAATCTATTGTTAG<br>ATTTCCTAATATTACAAACTTGTGCCCTTTTGGTGAAGTTTTAACGCCACCAGATTTGCATCTGTTTATGCTTGGAACAGGAAGAGAATCAGCAACTGTGTTGCTGATTATTCTGTCCTATATAATTCCGCATCATTTTCC<br>ACTTTTAAGTGTTATGGAGTGTCTCCTACTAAATTAAATGATCTCTGCTTTACTAATGTCTATGCAGATTCATTTGTAATTAGAGGTGATGAAGTCAGACAAATCGCTCCAGGGCAAACTGGAAAGATTGCTGATTATAA<br>TTATAAATTACCAGATGATTTTACAGGCTGCGTTATAGCTTGGAATTCTAACAATCTTGATTCTAAGGTTGGTGGTAATTATAATTACCTGTATAGATTGTTTAGGAAGTCTAATCTCAAACCTTTTG**AGAGAGATATTTC**<br>**AACTGAAATCTATCAGGCCGGTAGCA*A*ACCTTGTAATGGTGTTAAAGGTTTTAATT**GTTACTTTCCTTTACAATCATATGGTTTCCAACCCACTAATGGTGTTGGTTACCAACCATACAGAGTAGTAGTACTTTCTTTTGA<br>ACTTCTACATGCACCAGCAACTGTTTGTGGACCTAAAAAGTCTACTAATTTGGTTAAAAACAAATGTGTCAATTTCAACTTCAATGGTTTAACAGGCACAGGTGTTCTTACTGAGTCTAACAAAAAGTTTCTGCCTTTCCA<br>ACAATTTGGCAGAGACATTGCTGACACTACTGATGCTGTCCGTGATCCACAGACACTTGAGATTCTTGACATTACACCATGTTCTTTTGGTGGTGTCAGTGTTATAACACCAGGAACAAATACTTCTAACCAGGTTGCTG<br>TTCTTTATCAGGATGTTAACTGCACAGAAGTCCCTGTTGCTATTCATGCAGATCAACTTACTCCTACTTGGCGTGTTTATTCTACAGGTTCTAATGTTTTTCAAACACGTGCAGGCTGTTTACGACGTGTTATATTTGCTAG<br>CATAACCCCTTGGGGCCTCTAAACGGGTCTTGAGGGGTTTTTTGGTCATAGCTGTTTCCTGAGGGGGTACAAGCCCCTAGTGCCAATCGAGTTGCCCGGAACATCA
484Q_1mut	AAGGTAGCCTAATTTTTGCTTCAGCTTAATCGGTAAAGGTATAGTAAAACGACGGCCAGTTAATACGACTCACTATAGGGGTCGATTTATCAAACTTTAGAGTCCAACCAACAGAATCTATTGTTAGATTTCCTAATATT<br>ACAAACTTGTGCCCTTTTGGTGAAGTTTTAACGCCACCAGATTTGCATCTGTTTATGCTTGGAACAGGAAGAGAATCAGCAACTGTGTTGCTGATTATTCTGTCCTATATAATTCCGCATCATTTTCCACTTTTAAGTGTT<br>ATGGAGTGTCTCCTACTAAATTAAATGATCTCTGCTTTACTAATGTCTATGCAGATTCATTTGTAATTAGAGGTGATGAAGTCAGACAAATCGCTCCAGGGCAAACTGGAAAGATTGCTGATTATAATTATAAATTACCA<br>GATGATTTTACAGGCTGCGTTATAGCTTGGAATTCTAACAATCTTGATTCTAAGGTTGGTGGTAATTATAATTACCTGTATAGATTGTTTAGGAAGTCTAATCTCAAACCTTTTGA**GAGAGATATTTCAACTGAAATCTAT**<br>**CAGGCCGGTAGCA*A*ACCTTGTAATGGTGTTCAAGGTTTTAAT**TGTTACTTTCCTTTACAATCATATGGTTTCCAACCCACTAATGGTGTTGGTTACCAACCATACAGAGTAGTAGTACTTTCTTTTGAACTTCTACATGCA<br>CCAGCAACTGTTTGTGGACCTAAAAAGTCTACTAATTTGGTTAAAAACAAATGTGTCAATTTCAACTTCAATGGTTTAACAGGCACAGGTGTTCTTACTGAGTCTAACAAAAAGTTTCTGCCTTTCCAACAATTTGGCAG<br>AGACATTGCTGACACTACTGATGCTGTCCGTGATCCACAGACACTTGAGATTCTTGACATTACACCATGTTCTTTTGGTGGTGTCAGTGTTATAACACCAGGAACAAATACTTCTAACCAGGTTGCTGTTCTTTATCAGG<br>ATGTTAACTGCACAGAAGTCCCTGTTGCTATTCATGCAGATCAACTTACTCCTACTTGGCGTGTTTATTCTACAGGTTCTAATGTTTTTCAAACACGTGCAGGCTGTTTAAAACAGAACCAGCCACATGCTAGCATAACCC<br>CTTGGGGCCTCTAAACGGGTCTTGAGGGGTTTTTTGGTCATAGCTGTTTCCTGTCTTTTATGGACCTTGCAAGACTATCAGACATA

484STOP_1mut	TTATAAAAAACCACGTTAAAGTTCATGACCATCGCTACAGGCTCGTAAAACGACGGCCAGTTAATACGACTCACTATAGGGAAGCTGCTGCTAGACACTTTAGAGTCCAACCAACAGAATCTATTGTTAGATTTCCTAA<br>TATTACAAACTTGTGCCCTTTTGGTGAAGTTTTTAACGCCACCAGATTTGCATCTGTTTATGCTTGGAACAGGAAGAGAATCAGCAACTGTGTTGCTGATTATTCTGTCCTATATAATTCCGCATCATTTTCCACTTTTAAG<br>TGTTATGGAGTGTCTCCTACTAAATTAAATGATCTCTGCTTTACTAATGTCTATGCAGATTCATTTGTAATTAGAGGTGATGAAGTCAGACAAATCGCTCCAGGGCAAACTGGAAAGATTGCTGATTATAATTATAAATT<br>ACCAGATGATTTTACAGGCTGCGTTATAGCTTGGAATTCTAACAATCTTGATTCTAAGGTTGGTGGTAATTATAATTACCTGTATAGATTGTTTAGGAAGTCTAATCTCAAACCTTTTG**AGAGAGATATTTCAACTGAAA**<br>**TCTATCAGGCCGGTAGCA*A*ACCTTGTAATGGTGTTTAAGGTTTTAATT**GTTACTTTCCTTTACAATCATATGGTTTCCAACCCACTAATGGTGTTGGTTACCAACCATACAGAGTAGTAGTACTTTCTTTTGAACTTCTACA<br>TGCACCAGCAACTGTTTGTGGACCTAAAAAGTCTACTAATTTGGTTAAAAACAAATGTGTCAATTTCAACTTCAATGGTTTAACAGGCACAGGTGTTCTTACTGAGTCTAACAAAAAGTTTCTGCCTTTCCAACAATTTG<br>GCAGAGACATTGCTGACACTACTGATGCTGTCCGTGATCCACAGACACTTGAGATTCTTGACATTACACCATGTTCTTTTGGTGGTGTCAGTGTTATAACACCAGGAACAAATACTTCTAACCAGGTTGCTGTTCTTTAT<br>CAGGATGTTAACTGCACAGAAGTCCCTGTTGCTATTCATGCAGATCAACTTACTCCTACTTGGCGTGTTTATTCTACAGGTTCTAATGTTTTTCAAACACGTGCAGGCTGTTTACGCCATCCTACGTAAAACCTAGCATAA<br>CCCCTTGGGGCCTCTAAACGGGTCTTGAGGGGTTTTTTGGTCATAGCTGTTTCCTGGTCCCCCTTCCAACCTGGCAGGTTATCAAGAATGTTAGTTA
484E_2mut	TCGATTCAACATCGTTAGCGACATAATCTCTAGGTGCTTTCACAGGATGTAAAACGACGGCCAGTTAATACGACTCACTATAGGGTATCTCCACAGTGAGGTCCACTTTAGAGTCCAACCAACAGAATCTATTGTTAGA<br>TTTCCTAATATTACAAACTTGTGCCCTTTTGGTGAAGTTTTTAACGCCACCAGATTTGCATCTGTTTATGCTTGGAACAGGAAGAGAATCAGCAACTGTGTTGCTGATTATTCTGTCCTATATAATTCCGCATCATTTTCCA<br>CTTTTAAGTGTTATGGAGTGTCTCCTACTAAATTAAATGATCTCTGCTTTACTAATGTCTATGCAGATTCATTTGTAATTAGAGGTGATGAAGTCAGACAAATCGCTCCAGGGCAAACTGGAAAGATTGCTGATTATAAT<br>TATAAATTACCAGATGATTTTACAGGCTGCGTTATAGCTTGGAATTCTAACAATCTTGATTCTAAGGTTGGTGGTAATTATAATTACCTGTATAGATTGTTTAGGAAGTCTAATCTCAAACCTTTTGA**GAGAGATATTTCA**<br>**ACTGAAATCTATCAGGCCGGTA*A*CA*A*ACCTTGTAATGGTGTTGAAGGTTTTAAT**TGTTACTTTCCTTTACAATCATATGGTTTCCAACCCACTAATGGTGTTGGTTACCAACCATACAGAGTAGTAGTACTTTCTTTTGAA<br>CTTCTACATGCACCAGCAACTGTTTGTGGACCTAAAAAGTCTACTAATTTGGTTAAAAACAAATGTGTCAATTTCAACTTCAATGGTTTAACAGGCACAGGTGTTCTTACTGAGTCTAACAAAAAGTTTCTGCCTTTCCAA<br>CAATTTGGCAGAGACATTGCTGACACTACTGATGCTGTCCGTGATCCACAGACACTTGAGATTCTTGACATTACACCATGTTCTTTTGGTGGTGTCAGTGTTATAACACCAGGAACAAATACTTCTAACCAGGTTGCTGT<br>TCTTTATCAGGATGTTAACTGCACAGAAGTCCCTGTTGCTATTCATGCAGATCAACTTACTCCTACTTGGCGTGTTTATTCTACAGGTTCTAATGTTTTTCAAACACGTGCAGGCTGTTTAAAGTTTCCGCGGGTTGCACT<br>AGCATAACCCCTTGGGGCCTCTAAACGGGTCTTGAGGGGTTTTTTGGTCATAGCTGTTTCCTGGGAGATGTGGTAAAAATGCCGGTGGCTACTTC
484K_2mut	GAAACAGAATGCACATGTACATCGGCGACGGTTCGATTGCTGCTGTGTAAAACGACGGCCAGTTAATACGACTCACTATAGGGGTTTTATGCGCGCCAACACTTTAGAGTCCAACCAACAGAATCTATTGTTAGATTTC<br>CTAATATTACAAACTTGTGCCCTTTTGGTGAAGTTTTTAACGCCACCAGATTTGCATCTGTTTATGCTTGGAACAGGAAGAGAATCAGCAACTGTGTTGCTGATTATTCTGTCCTATATAATTCCGCATCATTTTCCACTTT<br>TAAGTGTTATGGAGTGTCTCCTACTAAATTAAATGATCTCTGCTTTACTAATGTCTATGCAGATTCATTTGTAATTAGAGGTGATGAAGTCAGACAAATCGCTCCAGGGCAAACTGGAAAGATTGCTGATTATAATTATA<br>AATTACCAGATGATTTTACAGGCTGCGTTATAGCTTGGAATTCTAACAATCTTGATTCTAAGGTTGGTGGTAATTATAATTACCTGTATAGATTGTTTAGGAAGTCTAATCTCAAACCTTTTG**AGAGAGATATTTCAACTG**<br>**AAATCTATCAGGCCGGTA*A*CA*A*ACCTTGTAATGGTGTTAAAGGTTTTAATT**GTTACTTTCCTTTACAATCATATGGTTTCCAACCCACTAATGGTGTTGGTTACCAACCATACAGAGTAGTAGTACTTTCTTTTGAACTTCT<br>ACATGCACCAGCAACTGTTTGTGGACCTAAAAAGTCTACTAATTTGGTTAAAAACAAATGTGTCAATTTCAACTTCAATGGTTTAACAGGCACAGGTGTTCTTACTGAGTCTAACAAAAAGTTTCTGCCTTTCCAACAAT<br>TTGGCAGAGACATTGCTGACACTACTGATGCTGTCCGTGATCCACAGACACTTGAGATTCTTGACATTACACCATGTTCTTTTGGTGGTGTCAGTGTTATAACACCAGGAACAAATACTTCTAACCAGGTTGCTGTTCTT<br>TATCAGGATGTTAACTGCACAGAAGTCCCTGTTGCTATTCATGCAGATCAACTTACTCCTACTTGGCGTGTTTATTCTACAGGTTCTAATGTTTTTCAAACACGTGCAGGCTGTTTAGTAGGGACTTACTCTAAGCTAGCA<br>TAACCCCTTGGGGCCTCTAAACGGGTCTTGAGGGGTTTTTTGGTCATAGCTGTTTCCTGTCGCCTGCGTGACTCATTTTACCATGCTAGATCGTCG
484Q_2mut	TGACAATTGTAGGCTGTGCCTCTTTCACCGGTAATCGGCGGGTAAAACGACGGCCAGTTAATACGACTCACTATAGGGCGAGATTTCTGAAGACTTTAGAGTCCAACCAACAGAATCTATTGTTAGATTTCCTAATATT<br>ACAAACTTGTGCCCTTTTGGTGAAGTTTTTAACGCCACCAGATTTGCATCTGTTTATGCTTGGAACAGGAAGAGAATCAGCAACTGTGTTGCTGATTATTCTGTCCTATATAATTCCGCATCATTTTCCACTTTTAAGTGTT<br>ATGGAGTGTCTCCTACTAAATTAAATGATCTCTGCTTTACTAATGTCTATGCAGATTCATTTGTAATTAGAGGTGATGAAGTCAGACAAATCGCTCCAGGGCAAACTGGAAAGATTGCTGATTATAATTATAAATTACCA<br>GATGATTTTACAGGCTGCGTTATAGCTTGGAATTCTAACAATCTTGATTCTAAGGTTGGTGGTAATTATAATTACCTGTATAGATTGTTTAGGAAGTCTAATCTCAAACCTTTTGA**GAGAGATATTTCAACTGAAATCTAT**<br>**CAGGCCGGTA*A*CA*A*ACCTTGTAATGGTGTTCAAGGTTTTAAT**TGTTACTTTCCTTTACAATCATATGGTTTCCAACCCACTAATGGTGTTGGTTACCAACCATACAGAGTAGTAGTACTTTCTTTTGAACTTCTACATGCA<br>CCAGCAACTGTTTGTGGACCTAAAAAGTCTACTAATTTGGTTAAAAACAAATGTGTCAATTTCAACTTCAATGGTTTAACAGGCACAGGTGTTCTTACTGAGTCTAACAAAAAGTTTCTGCCTTTCCAACAATTTGGCAG<br>AGACATTGCTGACACTACTGATGCTGTCCGTGATCCACAGACACTTGAGATTCTTGACATTACACCATGTTCTTTTGGTGGTGTCAGTGTTATAACACCAGGAACAAATACTTCTAACCAGGTTGCTGTTCTTTATCAGG<br>ATGTTAACTGCACAGAAGTCCCTGTTGCTATTCATGCAGATCAACTTACTCCTACTTGGCGTGTTTATTCTACAGGTTCTAATGTTTTTCAAACACGTGCAGGCTGTTTAACACCCACAGAGCACTGCTAGCATAACCCCT<br>TGGGGCCTCTAAACGGGTCTTGAGGGGTTTTTTGGTCATAGCTGTTTCCTGGGTTTACTTCGGGCTGGTATACTGGCGCGGGCCAGGGTTAGC
484STOP_2mut	GCACCTTCCTTGTCGAAAACCATAATGGTGTTCGTAAAACGACGGCCAGTTAATACGACTCACTATAGGGGGGCTTACCTCGGACTTTAGAGTCCAACCAACAGAATCTATTGTTAGATTTCCTAATATTACAAACTTGT<br>GCCCTTTTGGTGAAGTTTTTAACGCCACCAGATTTGCATCTGTTTATGCTTGGAACAGGAAGAGAATCAGCAACTGTGTTGCTGATTATTCTGTCCTATATAATTCCGCATCATTTTCCACTTTTAAGTGTTATGGAGTGT<br>CTCCTACTAAATTAAATGATCTCTGCTTTACTAATGTCTATGCAGATTCATTTGTAATTAGAGGTGATGAAGTCAGACAAATCGCTCCAGGGCAAACTGGAAAGATTGCTGATTATAATTATAAATTACCAGATGATTTT<br>ACAGGCTGCGTTATAGCTTGGAATTCTAACAATCTTGATTCTAAGGTTGGTGGTAATTATAATTACCTGTATAGATTGTTTAGGAAGTCTAATCTCAAACCTTTTG**AGAGAGATATTTCAACTGAAATCTATCAGGCCGG**<br>**TA*A*CA*A*ACCTTGTAATGGTGTTTAAGGTTTTAATT**GTTACTTTCCTTTACAATCATATGGTTTCCAACCCACTAATGGTGTTGGTTACCAACCATACAGAGTAGTAGTACTTTCTTTTGAACTTCTACATGCACCAGCAACT<br>GTTTGTGGACCTAAAAAGTCTACTAATTTGGTTAAAAACAAATGTGTCAATTTCAACTTCAATGGTTTAACAGGCACAGGTGTTCTTACTGAGTCTAACAAAAAGTTTCTGCCTTTCCAACAATTTGGCAGAGACATTGC<br>TGACACTACTGATGCTGTCCGTGATCCACAGACACTTGAGATTCTTGACATTACACCATGTTCTTTTGGTGGTGTCAGTGTTATAACACCAGGAACAAATACTTCTAACCAGGTTGCTGTTCTTTATCAGGATGTTAACTG<br>CACAGAAGTCCCTGTTGCTATTCATGCAGATCAACTTACTCCTACTTGGCGTGTTTATTCTACAGGTTCTAATGTTTTTCAAACACGTGCAGGCTGTTTACTGTTCCTGTATCCCTAGCATAACCCCTTGGGGCCTCTAAAC<br>GGGTCTTGAGGGGTTTTTTGGTCATAGCTGTTTCCTGCAGACGCTGGGACGTTTGTACCGTTTCTTATTCGTCCCG

Table S7. 484 Templates. Sequences (5’ → 3’) used to test the probes described in **Table S6**. The longest probe-binding region (corresponding to the energetic schemes without any NHRs) is highlighted in bold. The single-SNV differences between the targets are colored according to the scheme in **Figure 3** and described in **Table S8**. Single mutations used to evaluate probe robustness and taken from the SARS-CoV-2 Delta AY and Omicron spike sequences are denoted using italics. The first four templates have no mutations aside from that specified by the template name and colored accordingly. The next four have one mutation found in both Delta AY and Omicron lineages. The final four have an additional mutation found only in Omicron.

Template	Sequence
SARS-CoV-2N	ACCGGACCTTGCTTAACGCTAGCTTGGTAACGCGAGCACTCCGTTGCGGCGTAAAACGACGGCCAGTTAATACGACTCACTATAGGGGAGTACTGGAGGCGACGGGATATCGGTAATTATACAGTTTCCTGTTTACCT<br>TTTACAATTAATTGCCAGGAACCTAAATTGGGTAGTCTTGTAGTGCGTTGTTCGTTCTATGAAGACTTTTTAGAGTATCATGACGTTCGTGTTGTTTTAGATTTCATCTAAACGAACAAACTAAAATGTCTGATAATGGAC<br>CCCAAAATCAGCGAAATGCACCCCGCATTACGTTTGGTGGACCCTCAGATTCAACTGGCAGTAACCAGAATGGAGAACGCAGTGGGGCGCGATCAAAACAACGTCGGCCCCAAGGTTTACCCAATAATACTGCGTCTT<br>GGTTCACCGCTCTCACTCAACATGGCAAGGAAGACCTTAAATTCCCTCGAGGACAAGGCGTTCCAATTAACACCAATAGCAGTCCAGATGACCAAATTGGCTACTACCGAAGAGCTACCAGACGAATTCGTGGTGGTG<br>ACGGTAAAATGAAAGATCTCAGTCCAAGATGGTATTTCTACTACCTAGGAACTGGGCCAGAAGCTGGACTTCCCTATGGTGCTAACAAAGACGGCATCATATGGGTTGCAACTGAGGGAGCCTTGAATACACCAAAA<br>GATCACATTGGCACCCGCAATCCTGCTAACAATGCTGCAATCGTGCTACAACTTCCTCAAGGAACAACATTGCCAAAAGGCTTCTACGCAGAAGGGAGCAGAGGCGGCAGTCAAGCCTCTTCTCGTTCCTCATCACGTA<br>GTCGCAACAGTTCAAGAAATTCAACTCCAGGCAGCAGTAGGGGAACTTCTCCTGCTAGAATGGCTGGCAATGGCGGTGATGCTGCTCTTGCTTTGCTGCTGCTTGACAGATTGAACCAGCTTGAGAGCAAAATGTCTG<br>GTAAAGGCCAACAACAACAAGGCCAAACTGTCACTAAGAAATCTGCTGCTGAGGCTTCTAAGAAGCCTCGGCAAAAACGTACTGCCACTAAAGCATACAATGTAACACAAGCTTTCGGCAGACGTGGTCCAGAACAA<br>ACAGGTGTCTATACAAGTCCTAGCATAACCCCTTGGGGCCTCTAAACGGGTCTTGAGGGGTTTTTTGGTCATAGCTGTTTCCTGAATGGATCACAGCTATCTTTCAAGAGTGACAGAGGGACCGCG
SARS-CoV-2N1.1	CAGCACAAGGTAACGAAATTTCTCTATACTGAGGCCCCTTGGTCGTCGGGTAAAACGACGGCCAGTTAATACGACTCACTATAGGGCAAGTTAGTCTCATATCGGTAATTATACAGTTTCCTGTTTACCTTTTACAATTA<br>ATTGCCAGGAACCTAAATTGGGTAGTCTTGTAGTGCGTTGTTCGTTCTATGAAGACTTTTTAGAGTATCATGACGTTCGTGTTGTTTTAGATTTCATCTAAACGAACAAACTAAAATGTCTGATAATGGACCCCAAAATC<br>AGCGAAATGCACCCCGCATTACGTTTGGTGGACCCTCAGATTCAACTGGCAGTAACCAGAATGGAGAACGCAGTGGGGCGCGATCAAAACAACGTCGGCCCCAAGGTTTACCCAATAATACTGCGTCTTGGTTCACCG<br>CTCTCACTCAACATGGCAAGGAAGACCTTAAATTCCCTCGAGGACAAGGCGTTCCAATTAACACCAATAGCAGTCCAGATGACCAAATTGGCTACTACCGAAGAGCTACCAGACGAATTCGTGGTGGTGACGGTAAAA<br>TGAAAGATCTCAGTCCAAGATGGTATTTCTATTACCTAGGAACTGGGCCAGAAGCTGGACTTCCCTATGGTGCTAACAAAGACGGCATCATATGGGTTGCAACTGAGGGAGCCTTGAATACACCAAAAGATCACATTG<br>GCACCCGCAATCCTGCTAACAATGCTGCAATCGTGCTACAACTTCCTCAAGGAACAACATTGCCAAAAGGCTTCTACGCAGAAGGGAGCAGAGGCGGCAGTCAAGCCTCTTCTCGTTCCTCATCACGTAGTCGCAACA<br>GTTCAAGAAATTCAACTCCAGGCAGCAGTAGGGGAACTTCTCCTGCTAGAATGGCTGGCAATGGCGGTGATGCTGCTCTTGCTTTGCTGCTGCTTGACAGATTGAACCAGCTTGAGAGCAAAATGTCTGGTAAAGGCC<br>AACAACAACAAGGCCAAACTGTCACTAAGAAATCTGCTGCTGAGGCTTCTAAGAAGCCTCGGCAAAAACGTACTGCCACTAAAGCATACAATGTAACACAAGCTTTCGGCAGACGTGGTCCAGAACAAACAAAACAG<br>GCCCCTAGCATAACCCCTTGGGGCCTCTAAACGGGTCTTGAGGGGTTTTTTGGTCATAGCTGTTTCCTGTTACCAGAAAACCGCAGGCACCTCTTAACCCTACGAGTCTAC
SARS-CoV-2N1.2	AAGACAGTTCATAATAGGTATTGTGCCGAAACGGACTCTTCCGTAAAACGACGGCCAGTTAATACGACTCACTATAGGGTTACTTCCGAGGGACCTATAATATCGGTAATTATACAGTTTCCTGTTTACCTTTTACAATT<br>AATTGCCAGGAACCTAAATTGGGTAGTCTTGTAGTGCGTTGTTCGTTCTATGAAGACTTTTTAGAGTATCATGACGTTCGTGTTGTTTTAGATTTCATCTAAACGAACAAACTAAAATGTCTGATAATGGACCCCAAAAT<br>CAGCGAAATGCACCCCGCATTACGTTTGGTGGACCCTCAGATTCAACTGGCAGTAACCAGAATGGAGAACGCAGTGGGGCGCGATCAAAACAACGTCGGCCCCAAGGTTTACCCAATAATACTGCGTCTTGGTTCACC<br>GCTCTCACTCAACATGGCAAGGAAGACCTTAAATTCCCTCGAGGACAAGGCGTTCCAATTAACACCAATAGCAGTCCAGATGACCAAATTGGCTACTACCGAAGAGCTACCAGACGAATTCGTGGTGGTGACGGTAAA<br>ATGAAAGATCTCAGTCCAAGATGGTATTTCTACTACCTAGGAACTGGGCCAGAAGCTGCACTTCCCTATGGTGCTAACAAAGACGGCATCATATGGGTTGCAACTGAGGGAGCCTTGAATACACCAAAAGATCACATT<br>GGCACCCGCAATCCTGCTAACAATGCTGCAATCGTGCTACAACTTCCTCAAGGAACAACATTGCCAAAAGGCTTCTACGCAGAAGGGAGCAGAGGCGGCAGTCAAGCCTCTTCTCGTTCCTCATCACGTAGTCGCAAC<br>AGTTCAAGAAATTCAACTCCAGGCAGCAGTAGGGGAACTTCTCCTGCTAGAATGGCTGGCAATGGCGGTGATGCTGCTCTTGCTTTGCTGCTGCTTGACAGATTGAACCAGCTTGAGAGCAAAATGTCTGGTAAAGGC<br>CAACAACAACAAGGCCAAACTGTCACTAAGAAATCTGCTGCTGAGGCTTCTAAGAAGCCTCGGCAAAAACGTACTGCCACTAAAGCATACAATGTAACACAAGCTTTCGGCAGACGTGGTCCAGAACAAACCGTGGCT<br>AATTCGTTGGCTAGCATAACCCCTTGGGGCCTCTAAACGGGTCTTGAGGGGTTTTTTGGTCATAGCTGTTTCCTGGAACACCGGATCCTGCGCTTTTTAAGAATGT
SARS-CoV-2N2	GCTACCAACATCTTTAACTTCGTGTCATGCACACCTCGTGTGATCTTAGTAAAACGACGGCCAGTTAATACGACTCACTATAGGGCCGAGTCACCATATATCGGTAATTATACAGTTTCCTGTTTACCTTTTACAATTAAT<br>TGCCAGGAACCTAAATTGGGTAGTCTTGTAGTGCGTTGTTCGTTCTATGAAGACTTTTTAGAGTATCATGACGTTCGTGTTGTTTTAGATTTCATCTAAACGAACAAACTAAAATGTCTGATAATGGACCCCAAAATCAG<br>CGAAATGCACCCCGCATTACGTTTGGTGGACCCTCAGATTCAACTGGCAGTAACCAGAATGGAGAACGCAGTGGGGCGCGATCAAAACAACGTCGGCCCCAAGGTTTACCCAATAATACTGCGTCTTGGTTCACCGCT<br>CTCACTCAACATGGCAAGGAAGACCTTAAATTCCCTCGAGGACAAGGCGTTCCAATTAACACCAATAGCAGTCCAGATGACCAAATTGGCTACTACCGAAGAGCTACCAGACGAATTCGTGGTGGTGACGGTAAAATG<br>AAAGATCTCAGTCCAAGATGGTATTTCTATTACCTAGGAACTGGGCCAGAAGCTGCACTTCCCTATGGTGCTAACAAAGACGGCATCATATGGGTTGCAACTGAGGGAGCCTTGAATACACCAAAAGATCACATTGGC<br>ACCCGCAATCCTGCTAACAATGCTGCAATCGTGCTACAACTTCCTCAAGGAACAACATTGCCAAAAGGCTTCTACGCAGAAGGGAGCAGAGGCGGCAGTCAAGCCTCTTCTCGTTCCTCATCACGTAGTCGCAACAGTT<br>CAAGAAATTCAACTCCAGGCAGCAGTAGGGGAACTTCTCCTGCTAGAATGGCTGGCAATGGCGGTGATGCTGCTCTTGCTTTGCTGCTGCTTGACAGATTGAACCAGCTTGAGAGCAAAATGTCTGGTAAAGGCCAAC<br>AACAACAAGGCCAAACTGTCACTAAGAAATCTGCTGCTGAGGCTTCTAAGAAGCCTCGGCAAAAACGTACTGCCACTAAAGCATACAATGTAACACAAGCTTTCGGCAGACGTGGTCCAGAACAAACCTCCTCGGTCT<br>AGCTAGCATAACCCCTTGGGGCCTCTAAACGGGTCTTGAGGGGTTTTTTGGTCATAGCTGTTTCCTGATGCGTATCGACCCGCGCAATCACTATAGCGACATT
SARS-CoV-1N	TTGAAAACCCGTGGGAATATTGCCTTTCATCAGACTCTCTTGTAAAACGACGGCCAGTTAATACGACTCACTATAGGGCTTTTGATAATCCTGCTTACTATCAACTGTCAAGATCCAGCTGGTGGTGCGCTTATAGCTAG<br>GTGTTGGTACCTTCATGAAGGTCACCAAACTGCTGCATTTAGAGACGTACTTGTTGTTTTAAATAAACGAACAAATTAAAATGTCTGATAATGGACCCCAATCAAACCAACGTAGTGCCCCCCGCATTACATTTGGTGG<br>ACCCACAGATTCAACTGACAATAACCAGAATGGAGGACGCAATGGGGCAAGGCCAAAACAGCGCCGACCCCAAGGTTTACCCAATAATACTGCGTCTTGGTTCACAGCTCTCACTCAGCATGGCAAGGAGGAACTTA<br>GATTCCCTCGAGGCCAGGGCGTTCCAATCAACACCAATAGTGGTCCAGATGACCAAATTGGCTACTACCGAAGAGCTACCCGACGAGTTCGTGGTGGTGACGGCAAAATGAAAGAGCTCAGCCCCAGATGGTACTTC<br>TATTACCTAGGAACTGGCCCAGAAGCTTCACTTCCCTACGGCGCTAACAAAGAAGGCATCGTATGGGTTGCAACTGAGGGAGCCTTGAATACACCCAAAGACCACATTGGCACCCGCAATCCTAATAACAATGCTGCC<br>ACCGTGCTACAACTTCCTCAAGGAACAACATTGCCAAAAGGCTTCTACGCAGAGGGAAGCAGAGGCGGCAGTCAAGCCTCTTCTCGCTCCTCATCACGTAGTCGCGGTAATTCAAGAAATTCAACTCCTGGCAGCAGT<br>AGGGGAAATTCTCCTGCTCGAATGGCTAGCGGAGGTGGTGAAACTGCCCTCGCGCTATTGCTGCTAGACAGATTGAACCAGCTTGAGAGCAAAGTTTCTGGTAAAGGCCAACAACAACAAGGCCAAACTGTCACTAA<br>GAAATCTGCTGCTGAGGCATCTAAAAAGCCTCGCCAAAAACGTACTGCCACAAAACAGTACAACGTCACTCAAGCATTTGGGAGACGTGGTCCAGAACAAACCCAAGGAAATTTCGGGGACCAAGACCTACGGCTTT<br>GCCAGTCTTTCTAGCATAACCCCTTGGGGCCTCTAAACGGGTCTTGAGGGGTTTTTTGGTCATAGCTGTTTCCTGACCCAGCGCGCGGCAGGCTCGGAGGTGTAACTGGCTAAA
SARS-CoV-1N1.1	GTGTAAGCGATTATCACAAAAAATCTTCTCACGGCATTCAGAAAATTAGTGTAAAACGACGGCCAGTTAATACGACTCACTATAGGGCACAAGTGTTGGGAGTGTTACTATCAACTGTCAAGATCCAGCTGGTGGTGC<br>GCTTATAGCTAGGTGTTGGTACCTTCATGAAGGTCACCAAACTGCTGCATTTAGAGACGTACTTGTTGTTTTAAATAAACGAACAAATTAAAATGTCTGATAATGGACCCCAATCAAACCAACGTAGTGCCCCCCGCATT<br>ACATTTGGTGGACCCACAGATTCAACTGACAATAACCAGAATGGAGGACGCAATGGGGCAAGGCCAAAACAGCGCCGACCCCAAGGTTTACCCAATAATACTGCGTCTTGGTTCACAGCTCTCACTCAGCATGGCAAG<br>GAGGAACTTAGATTCCCTCGAGGCCAGGGCGTTCCAATCAACACCAATAGTGGTCCAGATGACCAAATTGGCTACTACCGAAGAGCTACCCGACGAGTTCGTGGTGGTGACGGCAAAATGAAAGAGCTCAGCCCCAG<br>ATGGTACTTCTACTACCTAGGAACTGGCCCAGAAGCTTCACTTCCCTACGGCGCTAACAAAGAAGGCATCGTATGGGTTGCAACTGAGGGAGCCTTGAATACACCCAAAGACCACATTGGCACCCGCAATCCTAATAA<br>CAATGCTGCCACCGTGCTACAACTTCCTCAAGGAACAACATTGCCAAAAGGCTTCTACGCAGAGGGAAGCAGAGGCGGCAGTCAAGCCTCTTCTCGCTCCTCATCACGTAGTCGCGGTAATTCAAGAAATTCAACTCCT<br>GGCAGCAGTAGGGGAAATTCTCCTGCTCGAATGGCTAGCGGAGGTGGTGAAACTGCCCTCGCGCTATTGCTGCTAGACAGATTGAACCAGCTTGAGAGCAAAGTTTCTGGTAAAGGCCAACAACAACAAGGCCAAAC<br>TGTCACTAAGAAATCTGCTGCTGAGGCATCTAAAAAGCCTCGCCAAAAACGTACTGCCACAAAACAGTACAACGTCACTCAAGCATTTGGGAGACGTGGTCCAGAACAAACCCAAGGAAATTTCGGGGACCAAGACC<br>TACGACGTGTTATATTTGCTAGCATAACCCCTTGGGGCCTCTAAACGGGTCTTGAGGGGTTTTTTGGTCATAGCTGTTTCCTGAGGGGGTACAAGCCCCTAGTGCCAATCGAGTTGCCCGGAACATCA
SARS-CoV-1N1.2	AAGGTAGCCTAATTTTTGCTTCAGCTTAATCGGTAAAGGTATAGTAAAACGACGGCCAGTTAATACGACTCACTATAGGGGTCGATTTATCAATTACTATCAACTGTCAAGATCCAGCTGGTGGTGCGCTTATAGCTAG<br>GTGTTGGTACCTTCATGAAGGTCACCAAACTGCTGCATTTAGAGACGTACTTGTTGTTTTAAATAAACGAACAAATTAAAATGTCTGATAATGGACCCCAATCAAACCAACGTAGTGCCCCCCGCATTACATTTGGTGG<br>ACCCACAGATTCAACTGACAATAACCAGAATGGAGGACGCAATGGGGCAAGGCCAAAACAGCGCCGACCCCAAGGTTTACCCAATAATACTGCGTCTTGGTTCACAGCTCTCACTCAGCATGGCAAGGAGGAACTTA<br>GATTCCCTCGAGGCCAGGGCGTTCCAATCAACACCAATAGTGGTCCAGATGACCAAATTGGCTACTACCGAAGAGCTACCCGACGAGTTCGTGGTGGTGACGGCAAAATGAAAGAGCTCAGCCCCAGATGGTACTTC<br>TATTACCTAGGAACTGGCCCAGAAGCTTGACTTCCCTACGGCGCTAACAAAGAAGGCATCGTATGGGTTGCAACTGAGGGAGCCTTGAATACACCCAAAGACCACATTGGCACCCGCAATCCTAATAACAATGCTGCC<br>ACCGTGCTACAACTTCCTCAAGGAACAACATTGCCAAAAGGCTTCTACGCAGAGGGAAGCAGAGGCGGCAGTCAAGCCTCTTCTCGCTCCTCATCACGTAGTCGCGGTAATTCAAGAAATTCAACTCCTGGCAGCAGT<br>AGGGGAAATTCTCCTGCTCGAATGGCTAGCGGAGGTGGTGAAACTGCCCTCGCGCTATTGCTGCTAGACAGATTGAACCAGCTTGAGAGCAAAGTTTCTGGTAAAGGCCAACAACAACAAGGCCAAACTGTCACTAA<br>GAAATCTGCTGCTGAGGCATCTAAAAAGCCTCGCCAAAAACGTACTGCCACAAAACAGTACAACGTCACTCAAGCATTTGGGAGACGTGGTCCAGAACAAACCCAAGGAAATTTCGGGGACCAAGACCTAAAACAGA<br>ACCAGCCACATGCTAGCATAACCCCTTGGGGCCTCTAAACGGGTCTTGAGGGGTTTTTTGGTCATAGCTGTTTCCTGTCTTTTATGGACCTTGCAAGACTATCAGACATA

SARS-CoV-1N2	TTATAAAAAACCACGTTAAAGTTCATGACCATCGCTACAGGCTCGTAAAACGACGGCCAGTTAATACGACTCACTATAGGGAAGCTGCTGCTAGACTTACTATCAACTGTCAAGATCCAGCTGGTGGTGCGCTTATAGC<br>TAGGTGTTGGTACCTTCATGAAGGTCACCAAACTGCTGCATTTAGAGACGTACTTGTTGTTTTAAATAAACGAACAAATTAAAATGTCTGATAATGGACCCCAATCAAACCAACGTAGTGCCCCCCGCATTACATTTGGT<br>GGACCCACAGATTCAACTGACAATAACCAGAATGGAGGACGCAATGGGGCAAGGCCAAAACAGCGCCGACCCCAAGGTTTACCCAATAATACTGCGTCTTGGTTCACAGCTCTCACTCAGCATGGCAAGGAGGAACT<br>TAGATTCCCTCGAGGCCAGGGCGTTCCAATCAACACCAATAGTGGTCCAGATGACCAAATTGGCTACTACCGAAGAGCTACCCGACGAGTTCGTGGTGGTGACGGCAAAATGAAAGAGCTCAGCCCCAGATGGTACT<br>TCTACTACCTAGGAACTGGCCCAGAAGCTTGACTTCCCTACGGCGCTAACAAAGAAGGCATCGTATGGGTTGCAACTGAGGGAGCCTTGAATACACCCAAAGACCACATTGGCACCCGCAATCCTAATAACAATGCTG<br>CCACCGTGCTACAACTTCCTCAAGGAACAACATTGCCAAAAGGCTTCTACGCAGAGGGAAGCAGAGGCGGCAGTCAAGCCTCTTCTCGCTCCTCATCACGTAGTCGCGGTAATTCAAGAAATTCAACTCCTGGCAGCA<br>GTAGGGGAAATTCTCCTGCTCGAATGGCTAGCGGAGGTGGTGAAACTGCCCTCGCGCTATTGCTGCTAGACAGATTGAACCAGCTTGAGAGCAAAGTTTCTGGTAAAGGCCAACAACAACAAGGCCAAACTGTCACT<br>AAGAAATCTGCTGCTGAGGCATCTAAAAAGCCTCGCCAAAAACGTACTGCCACAAAACAGTACAACGTCACTCAAGCATTTGGGAGACGTGGTCCAGAACAAACCCAAGGAAATTTCGGGGACCAAGACCTACGCCA<br>TCCTACGTAAAACCTAGCATAACCCCTTGGGGCCTCTAAACGGGTCTTGAGGGGTTTTTTGGTCATAGCTGTTTCCTGGTCCCCCTTCCAACCTGGCAGGTTATCAAGAATGTTAGTTA

Table S8. SARS-CoV-1/2 Robust Discrimination Templates. Sequences (5' → 3') ordered in pUC57 plasmids and used to test specific detection of SARS-CoV-1 and SARS-CoV-2 in the presence of one or two mutations. The probe-binding region is colored according to the scheme used in **Figure 3** (blue for SARS-CoV-1, red for SARS-CoV-2). Single mutations are highlighted using the opposite color, as the mutations in SARS-CoV-2 templates come from the SARS-CoV-1 sequence and mutations in the SARS-CoV-1 template come from the SARS-CoV-2 sequence. For each target, two single-mutation templates were constructed along with the exact match and a template with both mutations.

Probe	Sequence
SARS2	TCCAAGATGGTATTTCTACTACCTAGGAACTGGGCCAGAAGCTGGACTTCCCTATGGTG

SARS1	CCCAGATGGTACTTCTATTACCTAGGAACTGGCCCAGAAGCTTCACTTCCCTACGGC
417K	ACTGGAAAGATTGCTGATTATAATTATAAATTACCAGATGATTTTACAGGCTGCGTTATAGC
417N	CAAACTGGAAATATTGCTGATTATAATTATAAATTACCAGATGATTTTACAGGCTGCGTTATAGC
417T	CTGGAACGATTGCTGATTATAATTATAAATTACCAGATGATTTTACAGGCTGCGTTATAGC
452L	TTATAGCTTGGAATTCTAACAATCTTGATTCTAAGGTTGGTGGTAATTATAATTACCTGTATAGATTG
452R	TTATAGCTTGGAATTCTAACAATCTTGATTCTAAGGTTGGTGGTAATTATAATTACCGGTATAGAT
484E	TGAAATCTATCAGGCCGGTAGCACACCTTGTAATGGTGTTGAAGGTTTTAAT
484K	CTGAAATCTATCAGGCCGGTAGCACACCTTGTAATGGTGTTAAAGGTTTTAATT
484Q	TGAAATCTATCAGGCCGGTAGCACACCTTGTAATGGTGTTCAAGGTTTTAAT
484A	TGAAATCTATCAGGCCGGTAACAAACCTTGTAATGGTGTTGCAGGTTTTAAT
501N	AGGTTTTAATTGTTACTTTCCTTTACAATCATATGGTTTCCAACCCACTAATGGTGTTG
501Y	GGTTTTAATTGTTACTTTCCTTTACAATCATATGGTTTCCAACCCACTTATGGTGTTG
501Y-Om	AGGTTTTAATTGTTACTTTCCTTTACGATCATATAGTTTCCGACCCACTTATGGTGTT
501Y-OmBA.2	GGTTTTAATTGTTACTTTCCTTTACGATCATATGGTTTCCGACCCACTTATGGTGTT
614D	CCAGGAACAAATACTTCTAACCAGGTTGCTGTTCTTTATCAGGATGTTAACTGC
614G	CAGGAACAAATACTTCTAACCAGGTTGCTGTTCTTTATCAGGGTGTTAACTGC
MERS	GTGGCTTCTAATGATGCAATAATGACTCGTTGTTTAGCTATTCATTCTTGTTTTATAGAA
229E	GTGCAACCAACAGGGCCGGCCTATGTCATGATTGACAAAGTGGAGTTTGAAAATGGT
NL63	TTGTTAAAACAGCTGTTCAACCCACAGCTCCTGCATATGTTATTATTGATAAGGTAGATTTTGTTAATGGAT
OC43	CTTGGCAGGATTTGATATGTTAGATAATTATAAAGCCATTGATGTAGTACAGTATGAAGCTGATAGGAGAGCAT
OC43_1.2	GTATTGTAATGGAAGTATTGCATGTCAGTTCTGCTTGGCAGGATTTGATATGTTAGATAATTATAAAGCCATTG
OC43_2.1	TGGTTGATAAAAATCTGATAACTACTGCTAACACTGGTACGTCTGTTACAGAAACTATGTTTGATGTTTATGTG
HKU1	CTGATATTAAGCAAGTTGGTTGTATGATGCGTTTGTTCTATGATAGAGATGGACAGCGTGTTTACGA
FluA1	AAAGCCGAGATCGCGCAGAGACTTGAAGATGTCTTTGCTGGGAAAAACACAGATCTT
FluA2	TCAAAGCCGAGATCGCACAGAGACTGGAAAGTGTCTTTGCAGGAAAGAACACAGATCTT
FluB1	AACTTTGAAGCAGGAATTCTGGAGTGCTATGAAAGGTTTTCATGGCAAAGAGCCCTTGAC
FluB2	CCAATGCCACCATAAACTTCGAAGCAGGAATTCTGGAGTGCTATGAAAGACTTTCATGG

Table S9. Probe Seqs. Sequences (5’ → 3’) used for detection of all coronaviruses (and influenza A and B) targets. Sequences included in this table only reflect the portions of the viral genome used for detection and do not include proprietary sequences used for anchoring the probe to the surface.

Name	Sequence
SARSCoV-fP	GACCAAATTGGCTACTACCGA
SARSCoV-rP	ATTCAYGGCTCCCTCAGTTG
CoV2-D614G-fP	TGATGCTGTCCGTGATCCA
CoV2-D614G-rP	AGAATAAACACGCCAAGTAGGA
CoV2-E484KQ-N501Y-fP	CTCAAACCTTTTGAGAGAGATATTTCAAC

CoV2-E484KQ-N501Y-rP	GCTGGTGCATGTAGAAGTTCA
CoV2-K417NT-L452R-fP	GACAAATCGCTCCAGGG
CoV2-K417NT-L452R-rP	TCAAAAGGTTTGAGATTAGACTTCCTAA
CoV-NL63-fP	TGGTAATACTTTTATTAATGGTGATATTGC
CoV-NL63-rP	AAAGTCATACCGCCAAAAAGTG
CoV-229E-fP	GCACGTTTATAACACCCGAAG
CoV-229E-rP	CCAAAATGTTTCACAGGAATACAATC
CoV-HKU1-fP	AGCTTAAACGACCTGTAAATCCA
CoV-HKU1-rP	GAACAACTTTAACTTTAGAATGTAACAGAT
CoV-OC43-fP	TGTGATCTATATTCCATTCAGGATGT
CoV-OC43-rP	AGGGCGTAACTAACTATCAATTCA
MERS-CoV-fP	ACAGTGGGGTTATGTAGGCA
MERS-CoV-rP	CAATTTCTTTTCATGTGAGATATAAGGATAC
MS2-fP	GTGGGTCATCGTGGGGT
MS2-rP	GGTGACCTTTTGCAGGACTT
IPC-fP	CTACTCAGTTTATAAGACCGCCA
IPC-rP	GTTGGGAAGCCCGATTTGA
FluA-fP	TCTWACCGAGGTCGAAACGTA
FluA-rP	TTARCCATTCCATGAGAGCC
FluB1-fP	ACAAATCGAGGTGGGTCCC
FluB1-rP	TTTAGCCTGTTTAGGCGATCTT
FluB2-fP	ACAAATTGAGGTGGGTCCG
FluB2-rP	TTTAGTCTGTTTAGACGGTCTT

Table S10. Primer Seqs. Sequences (5’ → 3’) for all primers designed for the respiratory pathogen panel. Forward and reverse primers are designated by fP and rP, respectively, with the reverse primer in all cases responsible both for binding to the template RNA (and thereby mediating cDNA synthesis) and for synthesizing the strand used to displace the quencher arm on the probes. This allows for asymmetric RT-PCR to be used both for the reverse transcription and probe displacement.

Supplemental Figures

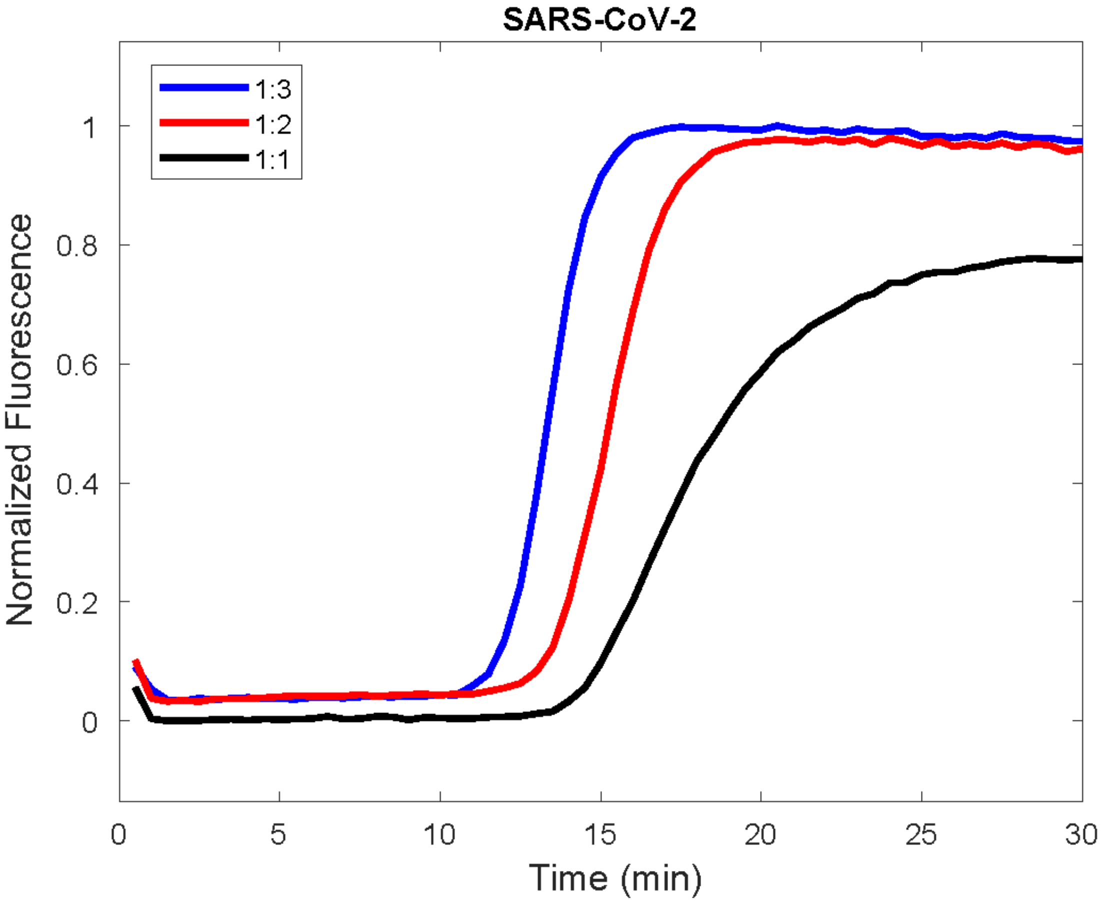

Figure S1. Primer Ratio. A set of experiments was performed using only SARS-CoV-1/2 primers in single-plex to assess the influence of the forward-to-reverse primer ratio (fP:rP) on toehold probe detection. The final forward primer concentration was held constant at 83 nM and the reverse primer concentration was tested at 250 nM, 166 nM, and 83 nM, corresponding to primer ratios of 1:3, 1:2, and 1:1, respectively. An input template concentration of 10,000 molecules of thermally released (at 75 °C for 5 minutes in a Benchmark MyBlock Mini Dry Bath heat block) SARS-CoV-2 Armored RNA Quant from Asuragen was used with standard RT-caPCR master mix composition and reaction conditions (10-minute RT-stage followed by 30-minute caPCR stage). We observe similar quencher displacement in response to either 1:3 or 1:2 primer ratios with an expected delay as the overall primer concentration is reduced. The 1:1

ratio produces significantly less sharp caPCR behavior with an additional delay in amplification. These results match our theoretical predictions, as using equal forward and reverse primer concentrations would produce equal amounts of the two strands of the amplicon. Within the annealing portion of the chamber (where the probes are localized), this translates to a reduced concentration of unbound ssDNA capable of displacing the quencher arm and thus detection kinetics that do not accurately reflect the progress of the reaction (as the displacing strand may also just bind to a free reverse complement in solution instead of finding the probe on the surface). By providing an excess of the primer that produces the displacing strand, there will still be available ssDNA beyond what could be bound by the reverse complement strand.

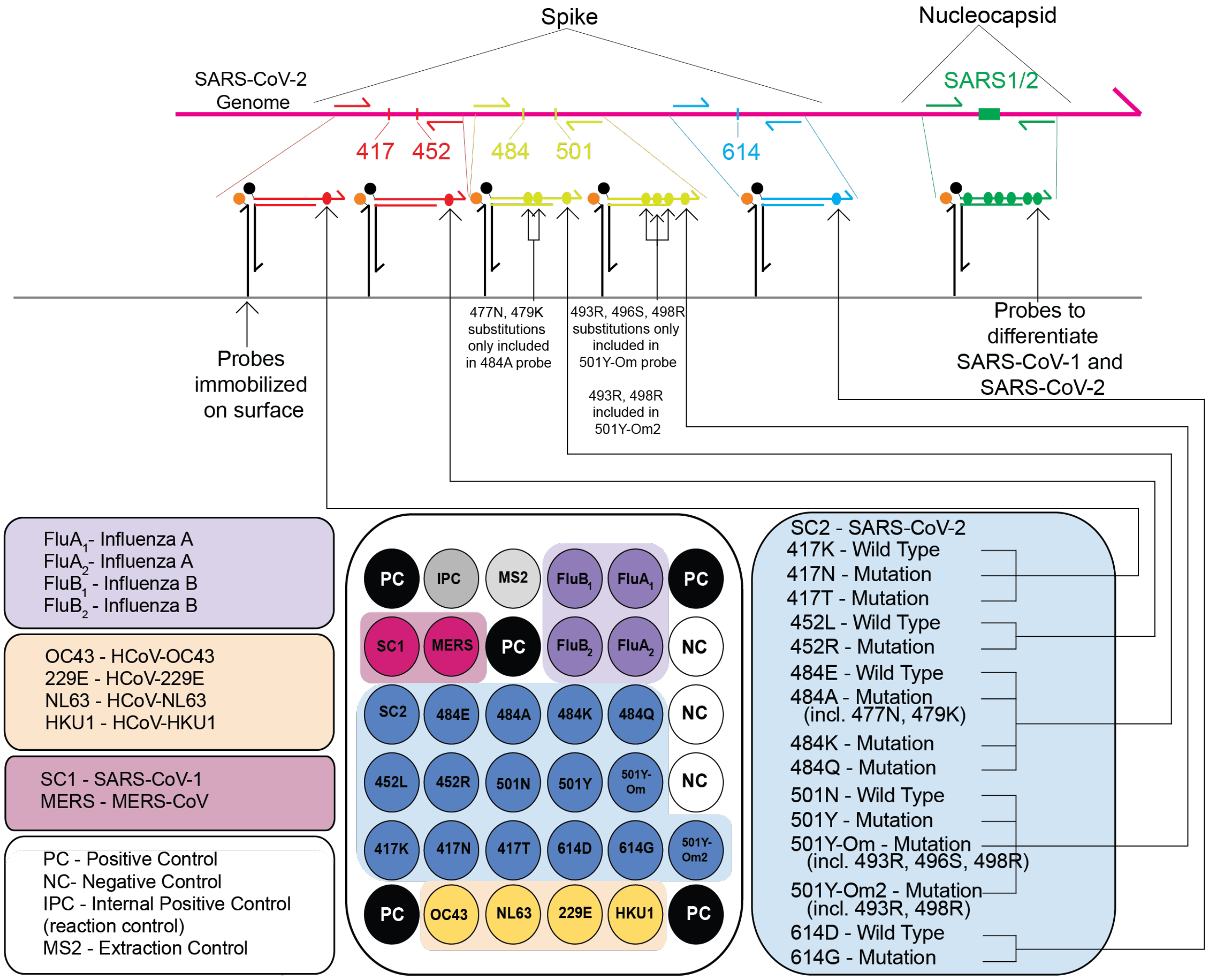

Figure S2. SARS-CoV-2 Probe Locations and Microarray Layout. Schematic of probe locations within the SARS-CoV-2 genome. 3 amplicons are used to capture 5 common mutation sites in the SARS-CoV-2 Spike sequence. The SNVs of interest are denoted with the ellipses in the toehold region of the probes. To account for the presence of non-target mutations, certain probes include other sequence changes, such as 477N and 479K in the 484A probe for the Omicron variants. These extra mutations are denoted by the ellipses in the domain (non-toehold) region of the probe.

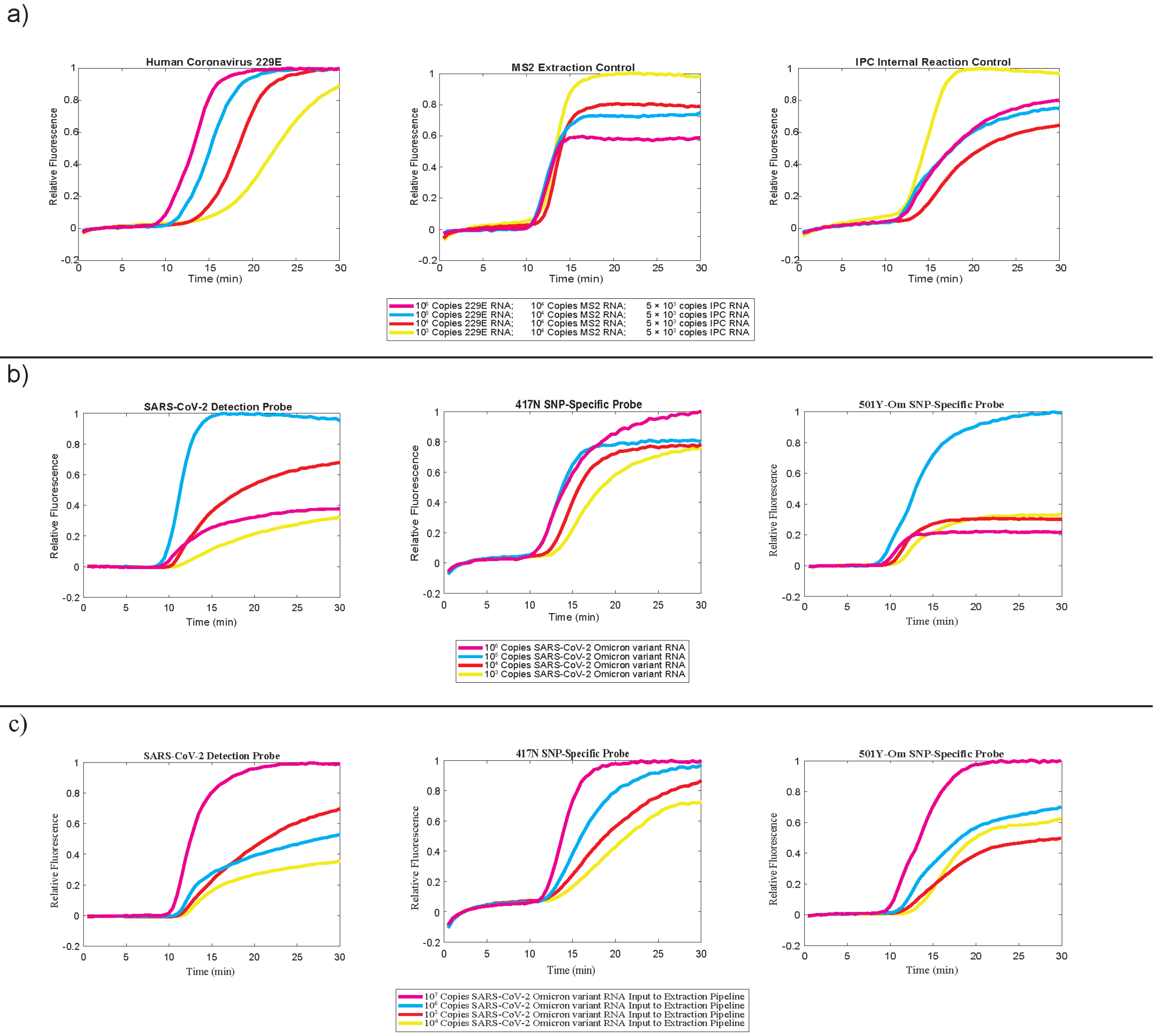

Figure S3. **Determining the limit of detection.** Limit of detection testing for SARS-CoV-2 and coronavirus 229E in the presence of controls. Titration of viral target RNA with MS2 and IPC RNAs fixed at 10,000 and 5,000 copies, respectively. a) HCoV-229E RNA; and b) SARS-CoV-2 Omicron RNA from 1,000 to 1,000,000 copies. c) SARS-CoV-2 Omicron RNA with input to extraction pipeline from 10,000 to 10,000,000 copies; input MS2 is 1,000,000. SARS-CoV-2 detection, 417N, and 501Y-Om probes are shown for Omicron results.

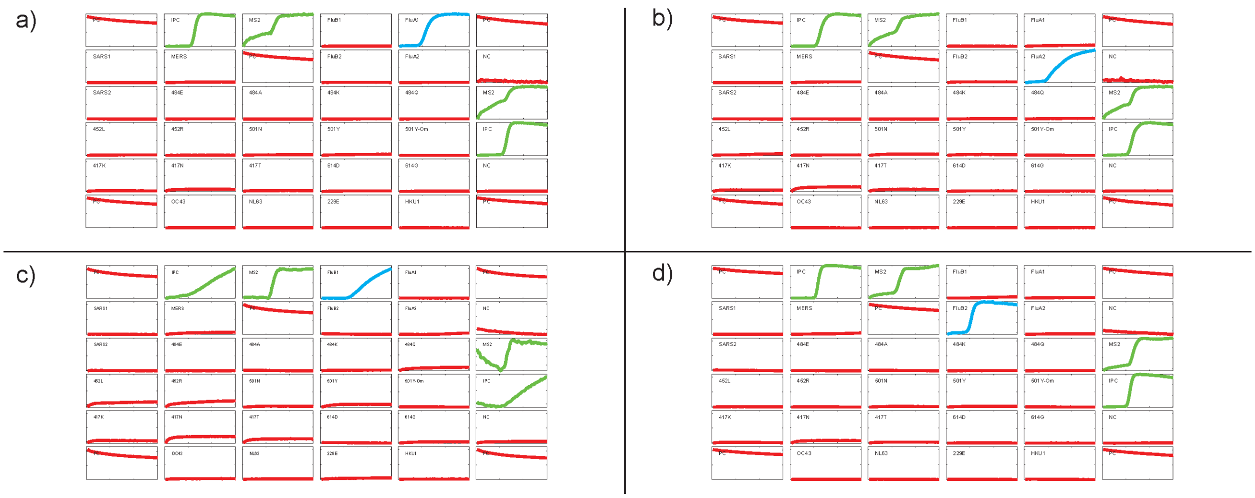

Figure S4. Detection of Influenza via RT-caPCR. By introducing three more primer sets targeting the sequence of matrix protein *M1* of influenza A and nonstructural protein *nsp1* in the Yamagata and Victoria lineages of influenza B, along with corresponding probes, we are able to extend our panel to include both coronaviruses and influenza. Influenza A detection is accomplished through two probes targeting slightly different sequences of the chosen gene, while influenza B targets the two distinct lineages in two probes. Here, we present results for 10^6 input molecules of: **a)** H3N2 RNA from Twist Bioscience; **b)** H1N1 RNA from Twist Bioscience; **c)** RNA from Yamagata lineage of influenza B from Twist Bioscience; and **d)** IVT-synthesized RNA from the Victoria lineage of influenza B. All runs were performed with all 14 primer sets, 5,000 molecules of IPC RNA, and 10,000 molecules of MS2 RNA. These results use pure RNA that has not been run through the extraction pipeline. We note that the panel layout used in

these runs was slightly different from those used for other presented data, as two of each control spot is present and the 501Y-Om2 probe is absent.

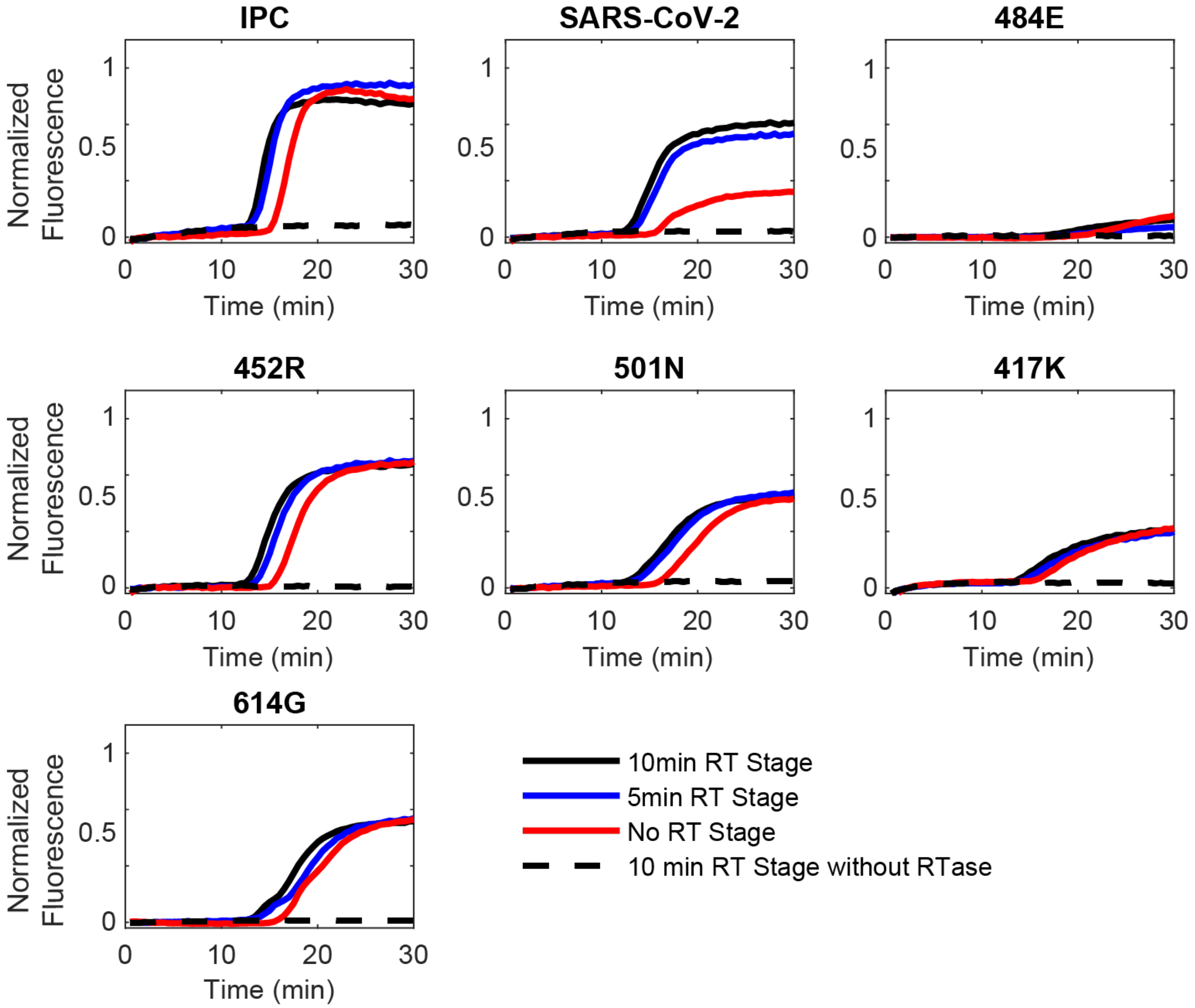

Figure S5. Different RT Times. Three runs were performed using 100,000 molecules of the SARS-CoV-2 Delta variant RNA and 5,000 molecules of IPC control RNA and varying the time of the 42 °C reverse transcription (RT) step from 10 minutes (the standard) down to 0 minutes (no dedicated RT step). A fourth run used the same input with a 10-minute RT stage but used a master mix lacking the reverse transcriptase (RTase) enzyme. All on-target curves are shown for all four reactions. 10-minute and 5-minute RT stages are broadly similar, suggesting that the overall reaction time can likely be reduced by reducing cDNA synthesis. Even without a

dedicated RT stage, some reverse transcription still occurs during the PCR stage, as the reverse transcriptase is still active. However, it is likely that completely forgoing the dedicated RT stage will compromise our limit of detection, so an optimal reaction protocol is likely between these two extremes. Even with the longest RT stage, a reaction lacking an RTase does not show any amplification, confirming that there is no effect of DNA contamination. Poor detection from the 484E probe is due to an unaccounted for SNV in the domain region.

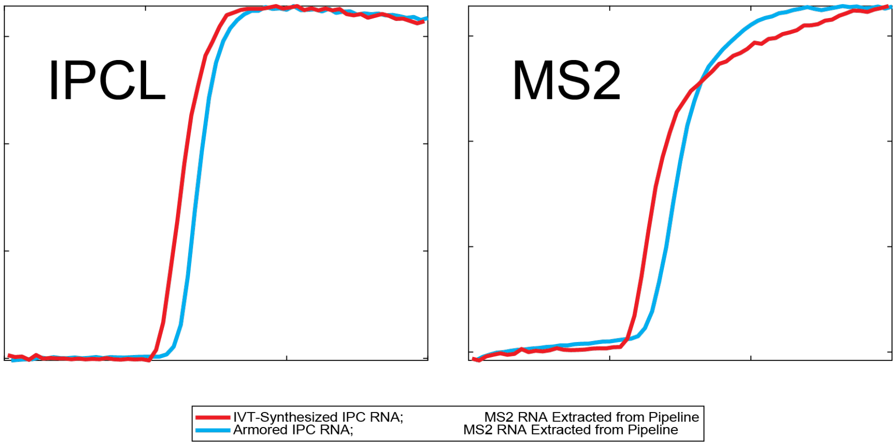

Figure S6. IPC RNA Source does not greatly affect amplification. Prior to optimizing our reaction conditions, we had been using Asuragen's Armored RNA technology as a source of our internal reaction control. We eventually moved to an IVT-synthesized template source to bring this part of our reaction into line with the viral RNA templates themselves, and to avoid the variability that is introduced by the thermal release stage necessary for removing the protective protein capsid around this RNA. We compared the performance of these two methods by running a negative control reaction, in which MS2 external control RNA was extracted from a VTM as described for all other experiments. IPC RNA was obtained from either 2.5 µL of thermally released 2000 molecules/µL Armored RNA or 2.5 µL 2000 molecules/µL IVT-synthesized IPC RNA. Thermal release was accomplished by treating the Armored RNA in a 0.6 mL tube for 5 minutes on a 65 °C heat block. The results from these two chips are overlaid and are broadly similar, with a slightly faster amplification with the IVT-synthesized RNA.
